# Xishaeleganins A–D, Sesquiterpenoid Hydroquinones from Xisha Marine Sponge *Dactylospongia elegans*

**DOI:** 10.3390/md20020118

**Published:** 2022-02-01

**Authors:** Bao Chen, Qingmin Zhao, Yu-Cheng Gu, Lefu Lan, Chang-Yun Wang, Yue-Wei Guo

**Affiliations:** 1 State Key Laboratory of Drug Research, Shanghai Institute of Materia Medica, Chinese Academy of Sciences, 555 Zu Chong Zhi Road, Zhangjiang Hi-Tech Park, Shanghai 201203, China; chenbao2333@163.com (B.C.); s19-zhaoqingmin@simm.ac.cn (Q.Z.); 2 Key Laboratory of Marine Drugs, The Ministry of Education of China, School of Medicine and Pharmacy, Ocean University of China, Qingdao 266003, China; 3 University of Chinese Academy of Sciences, No. 19A Yuquan Road, Beijing 100049, China; 4 Syngenta, Jealott’s Hill International Research Centre, Bracknell, Berkshire RG42 6EY, UK; yucheng.gu@syngenta.com; 5 Bohai Rim Advanced Research Institute for Drug Discovery, 198 Binhai East Road, High-Tech Zone, Yantai 264000, China

**Keywords:** sponge, *Dactylospongia elegans*, Thorectida, antibacterial, sesquiterpene hydroquinones

## Abstract

Four new sesquiterpene hydroquinones, xishaeleganins A–D (**6**–**9**), along with eleven known related ones (**12** and **14**–**23**) were isolated from the Xisha marine sponge *Dactylospongia elegans* (family Thorectida). Their structures were determined by extensive spectroscopic analysis, ECD calculations, and by comparison with the spectral data reported in the literature. Compounds **7**, **15**, **20**, and **21** showed significant antibacterial activity against *Staphylococcus aureus*, with minimum inhibitory concentration values of 1.5, 2.9, 5.6, and 5.6 µg/mL, which are comparable with those obtained for the positive control vancomycin (MIC: 1.0 µg/mL).

## 1. Introduction

**Figure 1:**
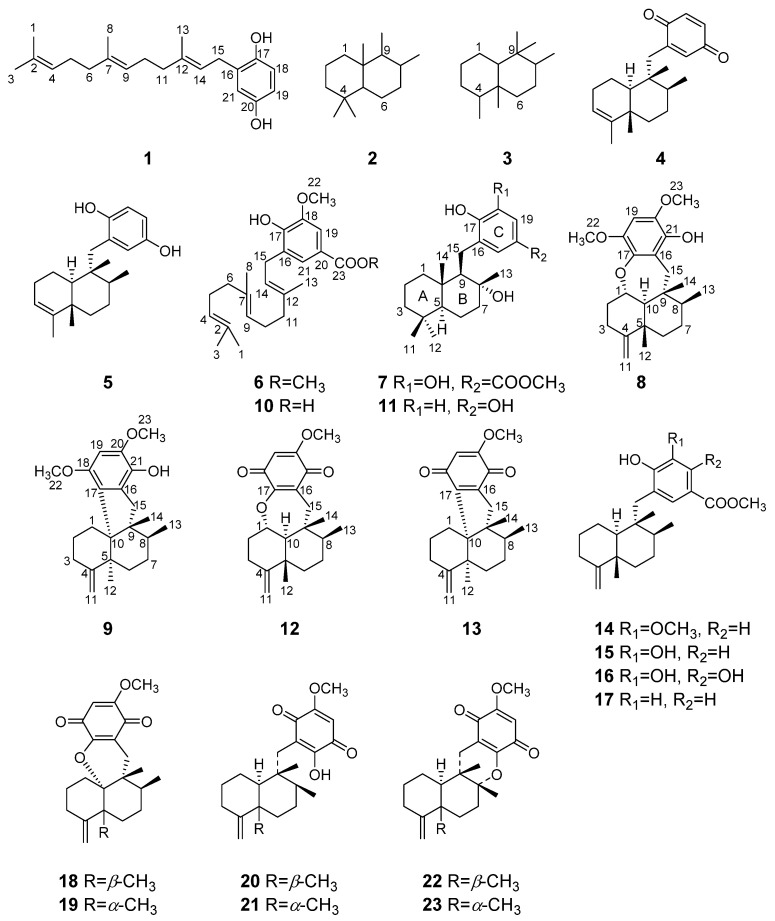
Structures of compounds **1**–**23**.

Metabolites of mixed sesquiterpene and quinone or hydroquinone biosynthesis are common to marine brown algae and sponges. In particular, these intriguing compounds are frequently discovered in the sponges of the genera *Fenestraspongia* [1], *Dysidea* [2], *Spongia* [3], *Hyrtios* [4], *Dactylospongia* [5], and *Hippospongia* [6]. In the last decades, numerous examples of this structure class have been reported, including those where the sesquiterpene unit exists in an acyclic (e.g., **1**), bicyclic normal drimane skeleton (e.g., **2**), or a rearranged drimane skeleton (4,9-friedodrimane skeleton) as exemplified in **3** (Figure 1). In addition to this, structural variation can focus on the degree of oxygenation and substitution to the aromatic ring. A wide range of remarkable bioactivities have been reported for many of these compounds, such as antiviral [7], antiangiogenic [8], antibacterial [9], antitumor [10], anti-inflammatory [11], and differentiation-inducing activities [12]. Avarone (**4**) and avarol (**5**), isolated from the Mediterranean sponge *Dysidea avara* in 1974, were the first two members of sesquiterpene quinone/hydroquinone [13]. Both **4** and **5** exhibited significant antiviral activity against HIV-I, and their great pharmaceutical potential stimulated the continued interests of chemists and pharmacologists [14,15]. A number of avarol derivatives [16] and related compounds [17] exhibiting interesting activity in enzyme assays measuring inhibition of various functions of HIV-I reverse transcriptase were discovered.

As part of our ongoing project towards the isolation of biologically active compounds from Chinese marine benthic invertebrates [18,19,20], a batch of *Dactylospongia elegans* sponges was collected off the coast of Xisha Island, Hainan Province, China, and chemically investigated. *Dactylospongia elegans* sponges belonging to the family Thorectida (order Dictyoceratida, class Demospongiae, and phylum Porifera) are prolific sources of biologically active compounds spanning a wide range of structural classes, from meroterpenoids [21,22], steroids [23], to diterpenoids [24], etc. In this report, we describe the isolation of four new sesquiterpene hydroquinones, xishaeleganins A–D (**6**–**9**), along with eleven known related ones (**12** and **14**–**23**) from the titular animals.

## 2. Results and Discussion

The frozen animals were cut into pieces and exhaustively extracted with acetone. The Et_2_O soluble portion of the acetone extract was chromatographed repeatedly over silica gel, Sephadex LH-20, and RP-HPLC to yield pure compounds **6**–**9**, **12,** and **14**–**23** (Figure 1), respectively.

Among them, the known compounds were readily identified as dactyloquinone C (**12**) [25], polyfibrospongol A (**14**) [26], dictyoceratin A (**15**) [27], dictyoceratin B (**16**) [27], dictyoceratin C (**17**) [28], dactyloquinone A (**18**) [29], dactyloquinone B (**19**) [29], ilimaquinone (**20**) [30], epi-ilimaquinone (**21**) [31], dactyloquinone D (**22**) [25], and dactyloquinone E (**23**) [25], by comparing their NMR data and optical rotation values with those reported in the literature.

**Figure 2:**
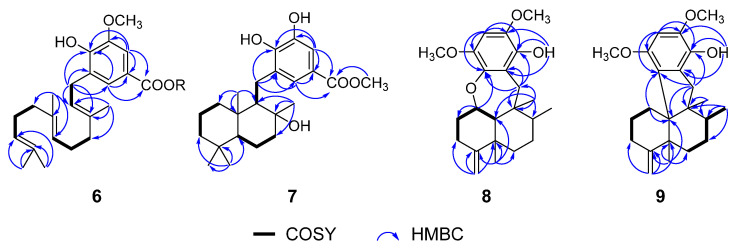
Selected key ^1^H-^1^H COSY and HMBC correlations of **6**–**9**.

Compound **6** was obtained as a stable yellow oil. Its molecular formula, C_24_H_34_O_4_, was established by HRESIMS (*m/z* 409.2357 [M + Na]^+^, calcd 409.2349). Its UV (*λ*_max_ 286 and 204 nm) and IR (*ν*_max_ 3414, 1709, 1609 cm^−1^) showed typical absorptions for hydroxyl, carbonyl group and aromatic ring, of which, the 1,2,3,5-tetrasubstituted pattern could be easily recognized by its ^1^H NMR data (Table 1), in particular meta-coupling between two aromatic protons of *δ*_H_ 7.41 (d, *J* = 1.9 Hz) and 7.52 (d, *J* = 1.9 Hz). Further, a polyene linear substituent assignable to a farnesyl (3,7,11-trimethyl-2,6,10-dodecatrienyl) moiety [5.32 (t, *J* = 7.4 Hz), 5.11 (t, *J* = 5.6 Hz), 5.07 (t, *J* = 5.6 Hz), 1.73 (s), 1.67 (s), 1.58 (2 s), 137.0 (C), 135.2 (C), 131.4 (C), 124.6 (CH), 124.3 (CH), 121.6 (CH), 25.8 (CH_3_), 17.7 (CH_3_), 16.4 (CH_3_), 16.1 (CH_3_)] was identified by the extensive analysis of ^1^H-^1^H COSY and HMBC spectrum (Figure 2). There are three ^1^H-^1^H COSY spin systems by clear correlations of H-4 (*δ*_H_ 5.11)/H_2_-5 (*δ*_H_ 2.09, 2.09)/H_2_-6 (*δ*_H_ 2.04, 1.95), H-9 (*δ*_H_ 5.07)/H_2_-10 (*δ*_H_ 2.04, 2.04)/H_2_-11 (*δ*_H_ 2.04, 1.95), and H-14 (*δ*_H_ 5.32)/H_2_-15 (*δ*_H_ 3.37, 3.37); these fragments were connected by the well-resolved HMBC correlations as shown in Figure 2. The hydroquinone part was located at C-15 (*δ*_C_ 28.1) by the HMBC correlations from H_2_-15 to C-16 (*δ*_C_ 127.4), C-17(*δ*_C_ 147.9) and C-21 (*δ*_C_ 124.6). A literature survey revealed that compound **6** showed great similarities with the model compound **10**, a sesquiterpene hydroquinone recently isolated from the fungus *Aspergillus flavipes* [32]. An overall comparison of the NMR data of **6** (Table 1; Table 2) and **10** revealed that the only difference between them happened at C-20 (the former carbomethoxy, the latter carboxylic acid), according to 14 mass units’ difference between their molecular weights. Compound **6** is simply a C-20 carboxyl methylation derivative of **10** and named xishaeleganin A.

Compound **7** was isolated as a stable, violet, amorphous solid. Its molecular formula of C_23_H_34_O_5_ was deduced by HRESIMS (*m/z* 413.2302 [M + Na]^+^, calcd 413.2298), implying seven degrees of unsaturation. The IR absorptions of *ν*_max_ 3401, 1687, 1595 cm^−1^, like those of **6**, indicated the presence of an aromatic ring and an ester carbonyl in the structure of **7**. Further, the diagnostic meta-coupling between two aromatic protons of *δ*_H_ 7.43 (d, *J* = 2.1 Hz) and 7.40 (d, *J* = 2.1 Hz) in the ^1^H NMR spectrum of **7** (Table 1) clearly suggested the presence of the same tetrasubstituted aromatic ring as that of co-occurring **15**. The one carbonyl carbon and an aromatic ring accounted for five out of the seven degrees of unsaturation, suggesting the presence of two rings in the remaining part of **7**. ^1^H NMR spectrum of **7** showed, bearing in mind the abovementioned two aromatic protons, five methyl signals (*δ*_H_ 3.85, s; 1.35, s; 0.94, s; 0.83, s; 0.79, s). The ^13^C NMR spectrum displayed resonances for one ester carbonyl carbon (*δ*_C_ 167.4), six aromatic carbons (*δ*_C_ 147.0, 145.1, 128.7, 125.2, 121.2, 113.7), three quaternary carbons (*δ*_C_ 40.1, 33.3 and an oxygenated one at *δ*_C_ 77.1), two methine carbons (*δ*_C_ 60.4, 56.0), six methylene carbons (*δ*_C_ 43.9, 41.7, 40.2, 27.5, 20.6, 18.3), and five methyl carbons (*δ*_C_ 33.4, 24.9, 21.5, 15.4 and an oxygenated one at *δ*_C_ 51.9).

**Figure 3:**
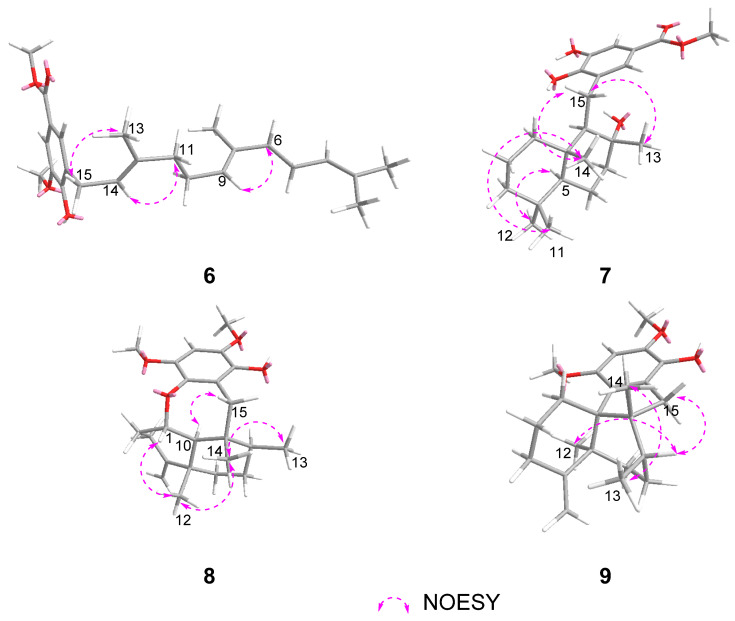
Selected key NOE correlations of **6**–**9**.

Subtraction of the already elaborated partial structure C from the molecular formula of **7** indicated the chemical composition of the remaining unassigned part of **7** is C_15_H_27_O, which must be of a bicyclic sesquiterpene nature, probably a drimane-type sesquiterpene. To confirm the hypothesis, we carefully studied the literature, finding that the ^1^H and ^13^C NMR data of **7** showed high similarity to those of model compound **11** (*ent*-yahazunol), a merosesquiterpene isolated from the sponge of the genus *Dysidea* collected in the Gulf of California [33]. In fact, **7** differs from **11** only at ring C, where the former is a 1,2,3,5-tetrasubstituted hydroquinone, while the latter is a 1,2,4-trisubstituted hydroquinone. In addition, the carbon signals at *δ*_C_ 128.7, 147.0, and 125.2 were located at the neighboring C-16, C-17, and C-21 positions by the HMBC correlations (Figure 2) from H_2_-15 to C-16 (*δ*_C_ 128.7)/C-17 (*δ*_C_ 147.0)/C-21 (*δ*_C_ 125.2). At the same time, both aromatic hydrogen protons displayed HMBC correlations with the methoxy carbonyl group (*δ*_C_ 167.4 and 51.9) which attached to the quaternary carbon (*δ*_C_ 121.2), allowing the carbon signals at *δ*_C_ 113.7, 121.2, and 125.2 to be assignable to C-19, C-20, and C-21 positions. In light of these observations, the planar structure of **7** was established as shown in Figure 2. Analogously to **11**, the relative configuration of **7** was elucidated to be the same as that of **11** by comparison of ^13^C NMR data of **7** with those of **11,** and confirmed by NOESY experiment as shown in Figure 3. Finally, considering the fact that all drimane sesquiterpenoids isolated from sponges possess the same absolute stereochemistry, and because the observed chemical shifts and coupling pattern of sesquiterpene moiety in **7** are similar to those of **11**, the absolute configuration (AC) of **7** is presumed to be identical to that of **11**.

The compounds **8** and **9** showed NMR data characteristic for a multisubstituted aromatic moiety, like that of co-occurring **6** and **7**. In particular, Compounds **8** and **9** not only shared a common pentasubstituted aromatic ring, but also possessed the same 4,9-friedodrimane skeleton, like that of co-occurring known sesquiterpene hydroquinones (**14**–**17**) as evidenced by a typical exo-cyclic olefin (*δ*_H_ 4.57, s; 4.57, s; *δ*_C_ 158.5, 103.4 in **8**. *δ*_H_ 4.67, s; 4.54, s; *δ*_C_ 157.9, 106.0 in **9**) and three high-field methyl signals (*δ*_H_ 0.93, s; 1.03, d, *J* = 6.6 Hz; 0.61, s; *δ*_C_ 21.8, 16.7, 15.4 in **8**. *δ*_H_ 0.81, s; 1.15, d, *J* = 7.3 Hz; 1.03, s; *δ*_C_ 24.3, 17.9, 24.8 in **9**). The structure elucidation of these new metabolites is described as follows.

Compound **8** was obtained as a violet amorphous solid. Its molecular formula, C_23_H_32_O_4_, was established by HRESIMS at *m*/*z* 373.2384 ([M + H]^+^; calcd 373.2373), suggesting eight degrees of unsaturation. Considering the already elaborated pentasubstituted aromatic ring and 4,9-friedodrimane motif, the remaining task is to assign the location of the substituents on the aromatic ring and the linking manner with the sesquiterpene unit. In fact, the ^1^H and ^13^C NMR data of **8** (Table 1; Table 2) were similar to those of co-occurring compound **12** (dactyloquinone C) except for the benzene portion, indicating the sesquiterpene moiety is connected to hydroquinone to form a seven-membered ring with an ether linkage between C-1 and C-17. Further, the key HMBC cross peaks from H-19 to C-17 (*δ*_C_ 142.0)/C-18 (*δ*_C_ 144.2)/C-20 (*δ*_C_ 142.2)/C-21 (*δ*_C_ 137.9), H_2_-15 to C-9 (*δ*_C_ 38.7)/C-16 (*δ*_C_ 122.9)/C-17 (*δ*_C_ 142.0)/C-21 (*δ*_C_ 137.9), and OH-21 to C-16 (*δ*_C_ 122.9)/C-20 (*δ*_C_ 142.2)/C-21 (*δ*_C_ 137.9) allowed the assignment of two methoxy groups at C-18 and C-20, and hydroxyl at C-21, completing the planar structure of **8**.

**Figure 4:**
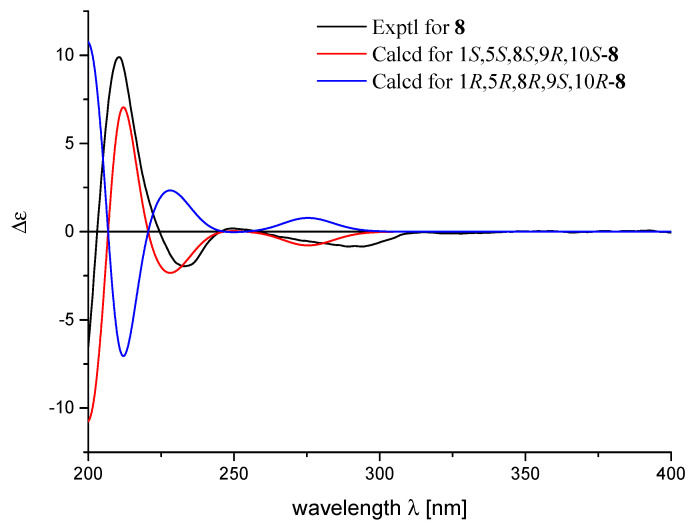
Experimental and calculated ECD spectra of **8**.

The relative configuration of **8** was deduced to be the same as those of **12** based on the biogenetic consideration, and supported by the NOESY correlations from H-1 to H_3_-12, from H_3_-14 to H_3_-12 and H_3_-13, and from H-10 to H_2_-15 as shown in Figure 3. Because the observed chemical shifts and coupling pattern of **8** are similar to those of **12**, the stereochemistry of **8** is presumed to be identical to that of **12**. The AC of **8** may consequently be considered to be 1*S*,5*S*,8*S*,9*R*,10*S* assuming **14** to be its precursor. To secure this assignment, the TDDFT-ECD method, which has proven to be a powerful and reliable method for the elucidation of the AC of natural products [34], was applied. As shown in Figure 4, the Boltzmann-averaged ECD spectrum of 1*S*,5*S*,8*S*,9*R*,10*S*-**8** was matched well to the experimental one of **8**. Consequently, the AC of compound **8** was determined as shown in Figure 1.

Compound **9** was also obtained as a violet amorphous solid. Its molecular formula, C_23_H_32_O_3_, was established by HREIMS at *m/z* 356.2346 (calcd 356.2346), 16 mass units less than that of **8**. Careful comparison of the ^13^C NMR data of **9** and **8** revealed the main differences between them happened at sesquiterpene moiety. In particular, the disappearance of the oxymethine carbon at *δ*_C_ 78.1 and methine carbon at *δ*_C_ 61.2 in **8**, accompanying the appearance of a methylene carbon at *δ*_C_ 32.1 and a quaternary carbon at *δ*_C_ 58.3 in **9**, indicated the sesquiterpene is connected to benzene moiety by a direct carbon-carbon linkage between the A/B ring junction C-10 and C-17 according to the degree of unsaturations inherent in its mass data.

**Figure 5:**
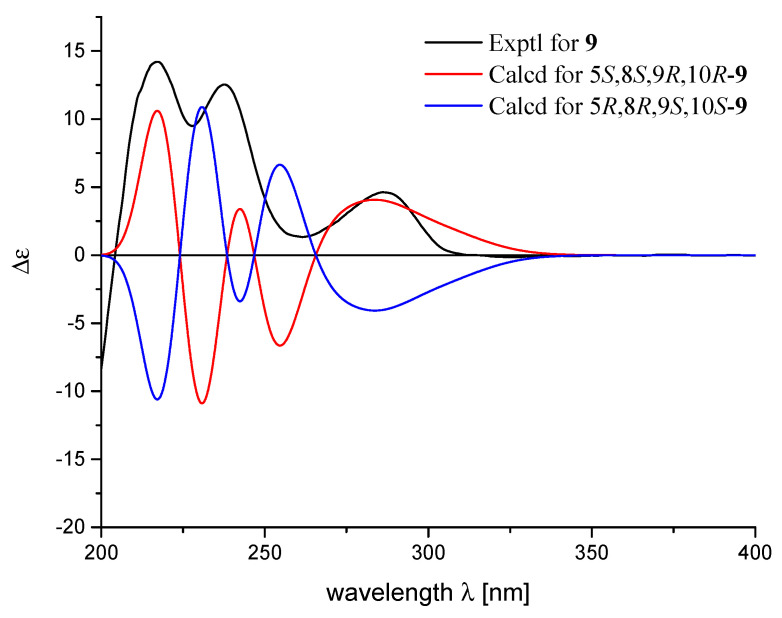
Experimental and calculated ECD spectra of **9**.

To confirm the deduced structure of **9**, 2D NMR spectra of **9** were analyzed in detail. A combination of HSQC and ^1^H−^1^H COSY data revealed two linear spin systems of shielded protons for **9** (Figure 1). Subsequently, extensive HMBC analysis, including the correlations of the conspicuous H_2_-11 *exo*-methylenes, the H_3_-13 and H_3_-14 methyls, and the H_2_-15 methylenes with neighboring carbons, supported a rearranged drimane sesquiterpene moiety identical to **14**–**17**. In this way, a nonprotonated carbon at *δ*_C_ 58.3 in the ^13^C NMR spectrum was assigned at the C-10 ring junction through its HMBC correlations with H_2_-1, H_3_-12 and H_2_-15. Due to the significant shifts of the hydroquinone carbons in the ^13^C NMR data from the other compounds, both the structure determination and NMR assignments of this portion of **9** were accomplished through HMBC analysis (Table 2). The long-range correlations with H_2_-15 were used to place the nonprotonated carbons *δ*_C_ 130.3, 131.3, and 135.3 at the neighboring C-16, C-17, and C-21 positions (Figure 1). An additional long-range correlation with H_2_-1 not only secured the signal at *δ*_C_ 131.3 at C-17 of the hydroquinone unit, but also directly linked this carbon at C-10 of the sesquiterpene moiety, thus constructing a five-membered ring corresponding to an additional degree of unsaturation (Figure 1). Accordingly, the other carbons at *δ*_C_ 130.3 and 135.3 were placed at C-16 and C-21, respectively. The HMBC correlations of the methine proton at *δ*_H_ 6.31 (*δ*_C_ 95.7) with C-17 C-18, C-20, and C-21, with no HMBC correlations with C-16 (*δ*_C_ 130.3), secured the remaining carbons signals *δ*_C_ 150.2, 95.7, and 144.8 at C-18, C-19, and C-20, respectively, fully assigning a pentasubstituted-hydroquinone moiety as depicted in Figure 2. A literature survey revealed that the spectroscopic data of **9** were reminiscent of those of model compound **13**, a sesquiterpene quinone previously reported from a *Dysidea* sp. sponge [34]. Overall comparison of the ^1^H and ^13^C NMR data of **9** and **13** revealed the significant differences occurred only in the hydroquinone portion (Table 1; Table 2) indicating the ACs at C-5, C-8, C-9, and C-10 in **9** should be the same as those of **13** [34]. Once again, by analog to **8**, the TDDFT-ECD approach was employed. The experimental ECD spectrum of **9** matched well with the calculated ECD spectrum of 5*S*,8*S*,9*R*,10*R*-**9** (Figure 5) and further supported its absolute configuration.

A literature survey revealed that the sesquiterpene quinones/hydroquinones show interesting antibacterial activity [9]; consequently, all the isolated compounds were tested for antibacterial activity against *Staphylococcus aureus*, *Streptococcus pyogenes*, and *Enterococcus faecium* (Table 3). Compounds **7**, **15**, **20**, and **21** showed maximum antibacterial activity against *S. aureus* (MIC: 1.5, 2.9, 5.6, and 5.6 µg/mL), comparable with that obtained for the positive control vancomycin (MIC: 1.0 µg/mL). As for *S. pyogenes*, compounds **7**, **8**, **12**, **15**, **16**, **20**, and **21** showed significant antibacterial activity with the MIC 1.5, 5.6, 2.8, 2.9, 1.5, 2.8, and 2.8 µg/mL, respectively. At the same time, compounds **7**, **8**, **12, 15**, and **16** showed significant antibacterial activity against *E. faecium* with the MIC 3.0, 5.6, 5.6, 1.4, and 3.0 µg/mL, respectively. These results highly suggested that the sesquiterpene hydroquinones/quinones such as compounds **7** and **15** have the potential to be the new lead compounds of antibiotics.

## 3. Materials and Methods

### 3.1. General Experimental Procedures

IR spectrum was recorded on a Nicolet 6700 spectrometer (Thermo Scientific, Waltham, MA, USA). Optical rotation was measured on a PerkinElmer 241 MC polarimeter (PerkinElmer, Fremont, CA, USA). ^1^H and ^13^C NMR spectra were acquired on a Bruker DRX-500 spectrometer (Bruker Biospin AG, Fällanden, Germany). Chemical shifts (*δ*) are reported in ppm with reference to the solvent signals, and coupling constants (*J*) are in Hz. ESIMS spectra were recorded on a Finngan-MAT-95 mass spectrometer and HRESIMS spectra were recorded on an Agilent 1290–6545 UHPLC-QTOF mass spectrometer. Commercial silica gel (Qingdao Haiyang Chemical Co., Ltd., Qingdao, China, 200–300 and 300–400 mesh) and Sephadex LH-20 gel (Amersham Biosciences, Little Chalfont, UK) were used for column chromatography (CC). Precoated silica gel GF-254 plates (Sinopharm Chemical Reagent Co., Shanghai, China) were used for analytical TLC. Reversed-phase (RP) HPLC was performed on an Agilent 1260 series liquid chromatograph equipped with a DAD G1315D detector at 210 nm (Agilent, Santa Clara, CA, USA). An Agilent semipreparative XDB-C18 column (5 μm, 250 × 9.4 mm) was employed for the purification. All solvents used for CC and HPLC were of analytical grade (Shanghai Chemical Reagents Co., Ltd., Shanghai, China) and chromatographic grade (Dikma Technologies Inc., Lake Forest, CA, USA), respectively.

### 3.2. Biological Material

The sponges of *D. elegans* was collected from the coast of Xisha Island, Hainan Province, China, in 2019, and identified by Dr. L.G. Yao (Shanghai Institute of Materia Medica, Chinese Academy of Sciences, Shanghai, China). A voucher specimen (No. 19-XS-39) is available for inspection at the Shanghai Institute of Materia Medica, CAS.

### 3.3. Extraction and Isolation

The frozen animals (275 g, dry weight) were cut into pieces and extracted exhaustively with acetone at room temperature (4 × 3.5 L). The organic extract was evaporated to give a brown residue, which was then partitioned between Et_2_O and H_2_O. The upper layer was concentrated under reduced pressure to give a Et_2_O portion (10.7 g). The Et_2_O extract was separated into eleven fractions (A–K) by gradient silica-gel column chromatography. The resulting fractions were then fractionated into sub-fractions by Sephadex LH-20. Fraction EA was separated by semi-preparative HPLC (90% acetonitrile), yielding compound **6** (0.6 mg). Fraction DC was separated by semi-preparative HPLC (95% acetonitrile), yielding compound **8** (4.0 mg). Fraction FC was separated by semi-preparative HPLC (95% methanol), yielding compound **20** (2.4 mg) and **21** (5.8 mg). Fraction GC was separated by semi-preparative HPLC (95% methanol), yielding compounds **9** (0.4 mg) and **14** (3.4 mg). Fraction HB was separated by semi-preparative HPLC (95% methanol), yielding compounds **7** (0.9 mg), **16** (8.7 mg), and **17** (4.1 mg). Fraction JB was separated by semi-preparative HPLC (88% acetonitrile), yielding compounds **12** (1.6 mg), **18** (1.8 mg), and **19** (2,9 mg). Fraction JD was separated by silica gel column eluting with petroleum ether (PE)/Et_2_O (4:1, *v*/*v*) to give compound **15** (15.4 mg). Fraction KA was separated by semi-preparative HPLC (100% methanol) to give compound **22** (3.2 mg) and **23** (0.8 mg).

### 3.4. Spectroscopic Data of Compounds

Xishaeleganin A (**6**): yellow oil; [*α*]${}_{D}^{20}$ −3.6 (*c* 0.06, CH_3_OH); IR (KBr): *ν*_max_ 3414, 2926, 2864, 1709, 1609, 1438, 1379, 1304, 1217, 1086, 1018, 803 cm^−1^; For ^1^H NMR (CDCl_3_, 500 MHz) and ^13^C NMR (CDCl_3_, 125 MHz) spectral data, see Table 1 and Table 2; HRESIMS *m/z* 409.2357 ([M + Na]^+^; calcd 409.2349).

Xishaeleganin B (**7**): violet amorphous solid; [*α*]${}_{D}^{20}$ −32.8 (*c* 0.09, CH_3_OH); IR (KBr): *ν*_max_ 3401, 2921, 2847, 1687, 1595, 1431, 1310, 1210, 1185, 1024, 1005, 768 cm^−1^; For ^1^H NMR (CDCl_3_, 500 MHz) and ^13^C NMR (CDCl_3_, 125 MHz) spectral data, see Table 1 and Table 2; HRESIMS *m/z* 413.2302 ([M + Na]^+^; calcd 413.2298).

Xishaeleganin C (**8**): violet amorphous solid; [*α*]${}_{D}^{20}$ −16.4 (*c* 0.40, CH_3_OH); IR (KBr): *ν*_max_ 3446, 2924, 2866, 1604, 1492, 1454, 1345, 1249, 1207, 1098, 1031, 973, 938, 890 cm^−1^; For ^1^H NMR (CDCl_3_, 500 MHz) and ^13^C NMR (CDCl_3_, 125 MHz) spectral data, see Table 1 and Table 2; HRESIMS *m/z* 373.2384 ([M + H]^+^; calcd 373.2373).

Xishaeleganin D (**9**): violet amorphous solid; [*α*]${}_{D}^{20}$ +174.6 (*c* 0.04, CH_3_OH); IR (KBr): *ν*_max_ 3452, 2953, 2924, 2860, 1627, 1591, 1498, 1460, 1377, 1338, 1293, 1092, 973, 887, 845 cm^−1^; For ^1^H NMR (CDCl_3_, 500 MHz) and ^13^C NMR (CDCl_3_, 125 MHz) spectral data, see Table 1 and Table 2; HREIMS *m/z* 356.2346 (calcd 356.2346).

### 3.5. Minimum Inhibitory Concentration (MIC) Determination

The MIC values for all antimicrobial agents were measured by 96-well micro-dilution method. Mueller–Hinton II broth (cation-adjusted, BD 212322) was used for MIC value determination. Generally, compounds were dissolved with DMSO to 20 mM as stock solutions. All samples were diluted with culture broth to 500 μM as the initial concentration. Further 1:2 serial dilutions were performed by addition of culture broth to reach concentrations ranging from 500 μM to 0.24 μM or lower. 100 µL of each dilution was distributed in 96-well plates, as well as sterile controls, growth controls (containing culture broth plus DMSO, without compounds), and positive controls (containing culture broth plus control antibiotics such as vancomycin). Each test and growth control well was inoculated with 5 µL of an exponential-phase bacterial suspension (about 10^5^ CFU/well). The 96-well plates were incubated at 37 °C for 24 h. MIC values of these compounds were defined as the lowest concentration to inhibit the bacterial growth completely. All MIC values were interpreted according to recommendations of the Clinical and Laboratory Standards Institute (CLSI).

## 4. Conclusions

**Scheme 1:**
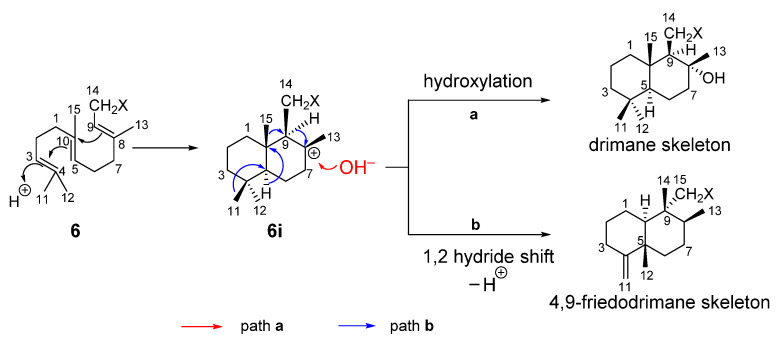
Plausible biosynthetic pathway proposed for drimane skeleton formation or rearranged drimane skeleton.

In conclusion, although the chemical studies on the South China Sea sponges *D. elegans* have been well documented in literature [5,21,22,23], the chemical reinvestigation of the same species collected from the same water area has still provided additional interesting and innovative results. The notable contribution towards the advance of meroterpenoid chemistry is the discovery of four new chemically diverse sesquiterpene hydroquinones, namely xishaeleganins A–D (**6**–**9**). It is interesting to note that although the structures of **6**–**9** are formally quite different, they are actually biogenetically related. It is obvious that compound **6** should be the common precursor for all sesquiterpene hydroquinones/quinones [35]. As outlined in Scheme 1, a plausible biogenetic pathway could be proposed to explain the biosynthesis of drimane skeleton of **7**. Thus, the cyclization of farnesyl in **6** takes place by an initial electrophilic attack at the head position of **6,** giving rise to a concerted process leading to a bicyclic carbocationic intermediate **6i**, which underwent further hydroxylation at C-8 to generate a drimane skeleton of **7**. Alternatively, starting from **6i**, other 4,9-friedodrimane-type sesquiterpene hydroquinones (e.g., **8**, **9**) are formed through concerted processes including 1,2 hydride shift accompanying the migration of methyls of C-4 and C-10. It is noteworthy that acyclic sesquiterpene quinones/hydroquinones are relatively rare, and to the best of our knowledge, until now, fewer than ten of this kind of metabolite were reported from sponges [35,36,37]. Last but not least, the recent report about the discovery of **10** from a fungus associated with mangrove *Acanthus ilicifolius* strongly suggests the microorganisms, which may be symbiotic, endophytic, or associated with the host sponge, could be the true producer of the sesquiterpene hydroquinones/quinones. Further study should be conducted to gain insight into the microorganisms responsible for producing sesquiterpene hydroquinones/quinones, and in particular to figure out the enzymatic mechanisms to biosynthesize these sponge metabolites.

## Figures and Tables

**Table 1:** ^1^H NMR Data for compounds **6**–**9**.

No.	6 ^a^	7 ^a^	8 ^a^	9 ^a^
	*δ*_H_ mult (*J* in Hz)	*δ*_H_ mult (*J* in Hz)	*δ*_H_ mult (*J* in Hz)	*δ*_H_ mult (*J* in Hz)
1	1.58, s	1.78, m; 0.67, m	3.89, m	1.98, m; 1.75, m
2		1.55, m; 1.40, m	2.47, m; 1.72, m	2.17, m; 1.70, m
3	1.67, s	1.31, m; 1.03, m	2.40, m; 2.20, m	2.50, m; 2.25, m
4	5.11, t (5.6)			
5	2.09, m; 2.09, m	0.93, m		
6	2.04, m; 1.95, m	1.71, m; 1.32, m	1.58, m; 1.58, m	2.25, m; 0.92, m
7		1.92, m; 1.61, m	1.56, m; 1.41, m	2.06, m; 1.35, m
8	1.58, s		1.42, m	1.89, m
9	5.07, t (5.6)	1.62, brd (6.1)		
10	2.04, m; 2.04, m		1.45, d (10.4)	
11	2.04, m; 1.95, m	0.79, s	4.57, s; 4.57, s	4.67, s; 4.54, s
12		0.83, s	0.93, s	0.81, s
13	1.73, s	1.35, s	1.03, d (6.6)	1.15, d (7.3)
14	5.32, t (7.4)	0.94, s	0.61, s	1.03, s
15	3.37, d (7.3); 3.37, d (7.3)	2.87, dd (15.2, 2.4); 2.52, dd (15.2, 6.1)	3.18, d (13.2); 2.36, d (13.2)	3.02, d (16.3); 2.39, d (16.3)
19	7.41, d (1.9)	7.43, d (2.1)	6.39, s	6.31, s
21	7.52, d (1.9)	7.40, d (2.1)		
22	3.93, s		3.81, s	3.59, s
23		3.85, s	3.84, s	3.85, s
24	3.87, s			
OH			5.24, s	5.08, s

^a^ Bruker DRX-500 spectrometer (500 MHz for ^1^H NMR) in CDCl_3_, chemical shifts (ppm) referred to CHCl_3_ (*δ*_H_ 7.26); assignments were deduced by analysis of 1D and 2D NMR spectra.

**Table 2:** ^13^C NMR Data for compounds **6**–**9**.

No.	6 ^a^	7 ^a^	8 ^a^	9 ^a^
	*δ*_C_ mult.	*δ*_C_ mult.	*δ*_C_ mult.	*δ*_C_ mult.
1	17.7, CH_3_	40.2, CH_2_	78.1, CH	32.1, CH_2_
2	131.4, C	18.3, CH_2_	36.2, CH_2_	24.0, CH_2_
3	25.8, CH_3_	41.7, CH_2_	31.4, CH_2_	32.2, CH_2_
4	124.3, CH	33.3, C	158.5, C	157.9, C
5	26.8, CH_2_	56.0, CH	40.4, C	43.5, C
6	39.8, CH_2_	20.6, CH_2_	37.6, CH_2_	31.4, CH_2_
7	135.2, C	43.9, CH_2_	28.3, CH_2_	26.2, CH_2_
8	16.4, CH_3_	77.1, C	41.9, CH	34.4, CH
9	124.6, CH	60.4, CH	38.7, C	50.8, C
10	26.9, CH_2_	40.1, C	61.2, CH	58.3, C
11	39.9, CH_2_	21.5, CH_3_	103.4, CH_2_	106.0, CH_2_
12	137.0, C	33.4, CH_3_	21.8, CH_3_	24.3, CH_3_
13	16.1, CH_3_	24.9, CH_3_	16.7, CH_3_	17.9, CH_3_
14	121.6, CH	15.4, CH_3_	15.4, CH_3_	24.8, CH_3_
15	28.1, CH_2_	27.5, CH_2_	35.8, CH_2_	43.4, CH_2_
16	127.4, C	128.7, C	122.9, C	130.0, C
17	147.9, C	147.0, C	142.0, C	131.3, C
18	146.1, C	145.1, C	144.2, C	150.2, C
19	109.6, CH	113.7, CH	96.9, CH	95.7, CH
20	121.5, C	121.2, C	142.2, C	144.8, C
21	124.6, CH	125.2, CH	137.9, C	135.3, C
22	56.4, CH_3_	167.4, C	58.0, CH_3_	55.6, CH_3_
23	167.3, C	51.9, CH_3_	56.5, CH_3_	56.3, CH_3_
24	52.0, CH_3_			

^a^ Bruker DRX-500 spectrometer (125 MHz for ^13^C NMR) in CDCl_3_, chemical shifts (ppm) referred to CHCl_3_ (*δ*_C_ 77.16); assignments were deduced by analysis of 1D and 2D NMR spectra.

**Table 3:** Antibacterial activities of compounds **7**–**23** (MIC µg/mL).

Compound	*S. aureus* USA300 LAC	*S. pyogenes* ATCC 12344	*E. faecium Efm-HS0649*
**7**	1.5	1.5	3.0
**8**	11.1	2.8	5.6
**9**	>186.0	11.6	>186.0
**12**	11.1	5.6	5.6
**15**	2.9	2.9	1.4
**16**	12.1	1.5	3.0
**18**	>178.0	44.5	22.2
**19**	178.0	22.2	22.2
**20**	5.6	2.8	11.2
**21**	5.6	2.8	11.2
**22**	>178.0	89.0	178.0
**23**	>178.0	22.2	178.0
Vancomycinn	1.0	0.25	>64.0

## Data Availability

Data are contained within the article or Supplementary Materials.

## Supplementary Materials

The following are available online at https://www.mdpi.com/article/10.3390/md20020118/s1, Figures S1−S4: 1D, 2D NMR, MS and IR spectra of compounds **6**–**9**; Figure S5: ^1^H and ^13^C NMR spectra of Compound **12** and **14**–**23**.
