# Supplementary material for: Xishaeleganins A–D, Sesquiterpenoid Hydroquinones from Xisha Marine Sponge Dactylospongia elegans
Source: Mar Drugs. 2022 Feb 1;20(2):118. doi: 10.3390/md20020118 (PMC8879793; doi:10.3390/md20020118)
Supplement: Supplementary file 1 [file marinedrugs-20-00118-s001.zip › marinedrugs-1582866-supplementary.pdf]

Xishaeleganins A–D, Sesquiterpenoid Hydroquinones from Xisha Marine Sponge *Dactylospongia elegans*

Bao Chen [1,2], Qingmin Zhao [1,3], Yu-Cheng Gu [4], Lefu Lan [1,3,*], Chang-Yun Wang [2,*] and Yue-Wei Guo [1,5,*]

1 State Key Laboratory of Drug Research, Shanghai Institute of Materia Medica, Chinese Academy of Sciences, 555 Zu Chong Zhi Road, Zhangjiang Hi-Tech Park, Shanghai, 201203, China; ywguo@simm.ac.cn; llan@simm.ac.cn

2 Key Laboratory of Marine Drugs, The Ministry of Education of China, School of Medicine and Pharmacy, Ocean University of China, Qingdao, 266003, China; changyun@ouc.edu.cn; chenbao2333@163.com

3 University of Chinese Academy of Sciences, No.19A Yuquan Road, Beijing 100049, P. R. China; s19-zhaoqingmin@simm.ac.cn

4 Bohai rim Advanced Research Institute for Drug Discovery, 198 Binhai East Road, High-tech Zone, Yantai, 264000, China; ywguo@simm.ac.cn

5 Syngenta, Jealott's Hill International Research Centre, Bracknell, Berkshire RG42 6EY, United Kingdom; yucheng.gu@syngenta.com

* Correspondence: llan@simm.ac.cn (L.F.L.); changyun@ouc.edu.cn (C.-Y.W.); ywguo@simm.ac.cn (Y.-W.G.)

Contents

Figure S1 1D, 2D NMR, MS and IR spectra of Compound 6 7

Figure S2 1D, 2D NMR, MS and IR spectra of Compound 7 12

Figure S3 1D, 2D NMR, MS and IR spectra of Compound 8 17

Figure S4 1D, 2D NMR, MS and IR spectra of Compound 9 22

Figure S5 ^{1}H and ^{13}C NMR spectra of Compound 12 and 14–23 33

Qualitative Analysis Report

**Data Filename**	ESIH202103582.d	**Sample Name**	A8-A8-3JCA31
**Sample ID**		**Position**	P1-C5
**Instrument Name**	Agilent G6520 Q-TOF	**Acq Method**	20160322_MS_ESIH_POS_1min.m
**Acquired Time**	7/23/2021 16:24:06	**IRM Calibration Status**	Success
**DA Method**	small molecular data analysis method.m	**Comment**	ESIH by zhuzhenyun

User Spectra

Fragmentor Voltage	Collision Energy	Ionization Mode
200	0	ESI

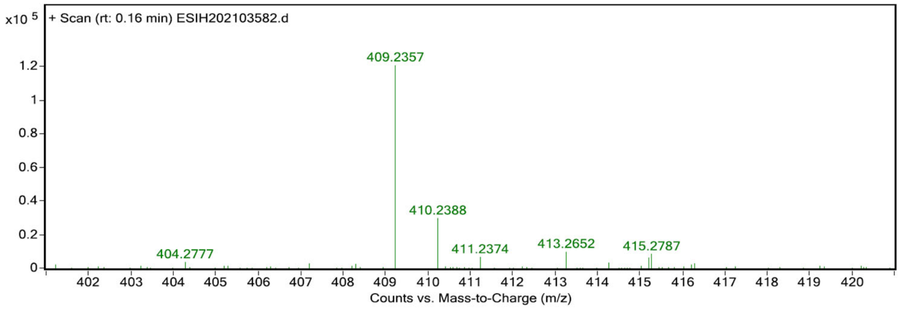

Formula Calculator Results

m/z	Calc m/z	Diff (mDa)	Diff (ppm)	Ion Formula	Ion
409.2357	409.2349	-0.76	-1.86	C24 H34 Na O4	(M+Na)+

--- End Of Report ---

Agilent Technologies Page 1 of 1 Printed at: 16:30 on: 7/23/2021

(a) HRESIMS spectrum of compound 6

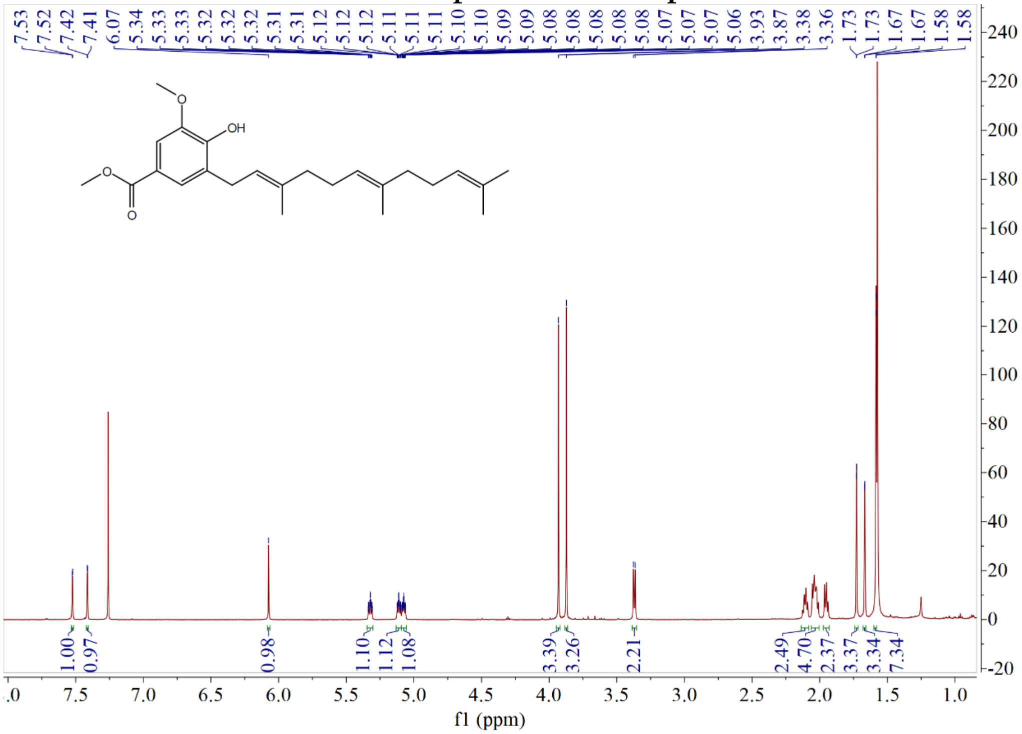

(b) ^{1}H NMR spectrum of compound 6 in $CDCl_3$

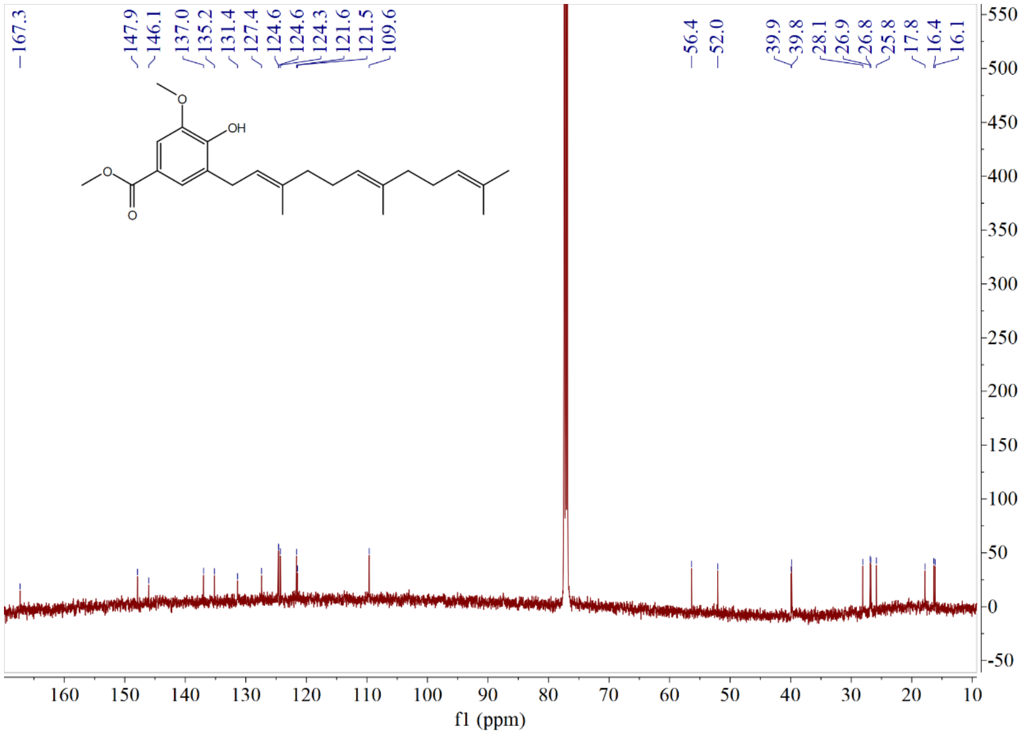

(c) ^{13}C NMR spectrum of compound 6 in $CDCl_3$

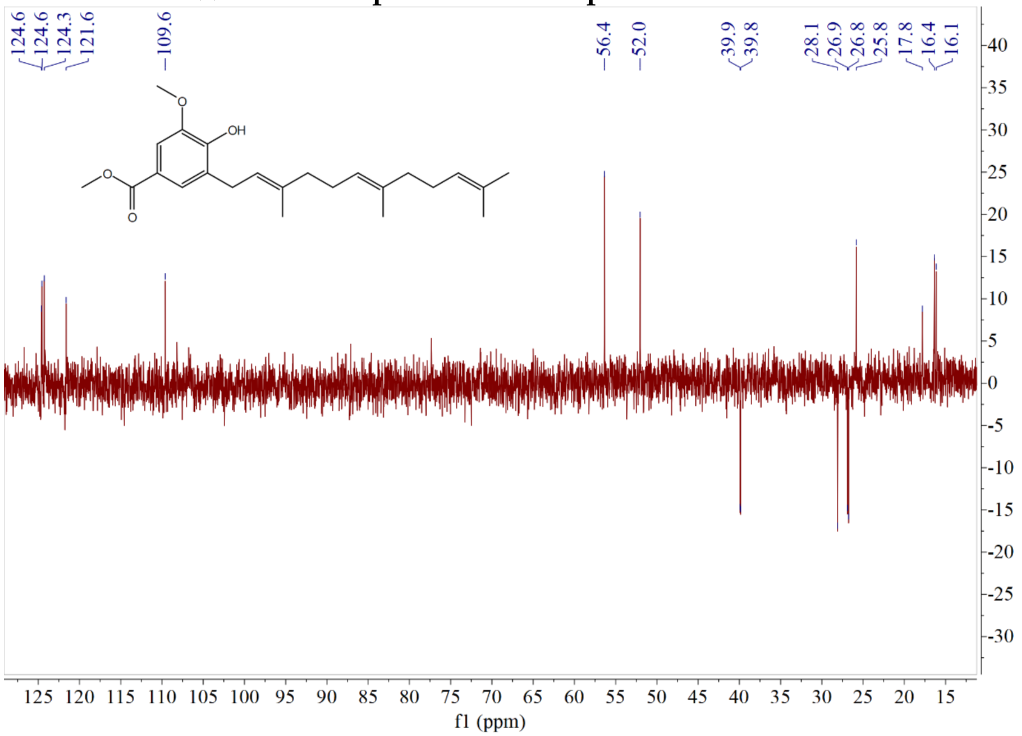

(d) DEPT spectrum of compound 6 in $CDCl_3$

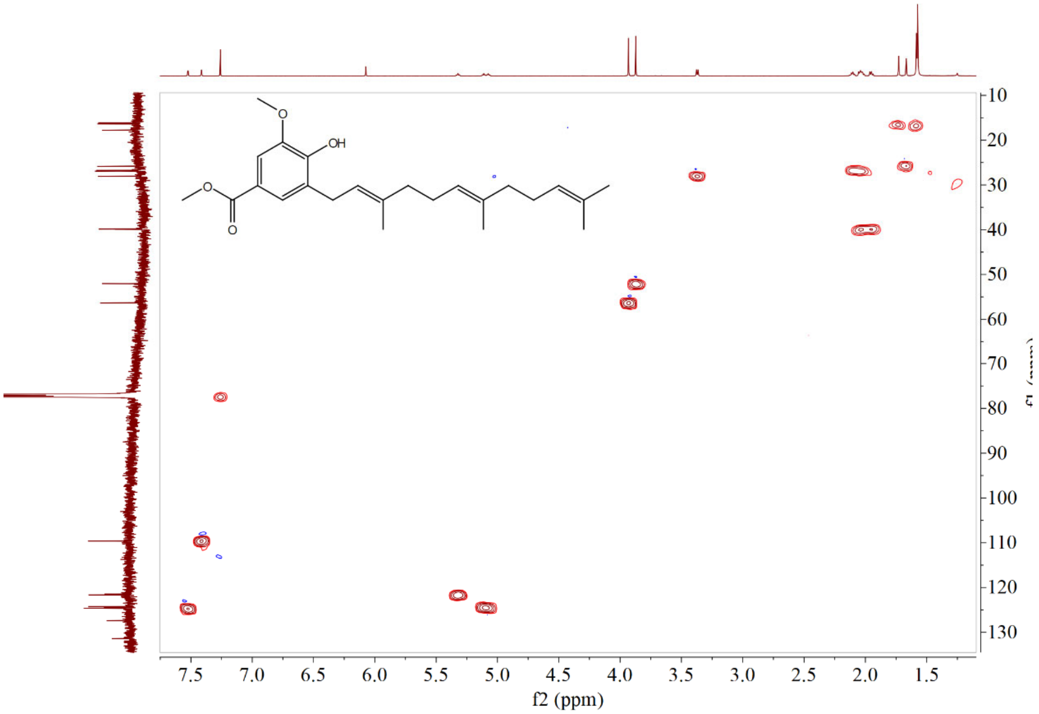

(e) HSQC spectrum of compound 6 in CDCl₃

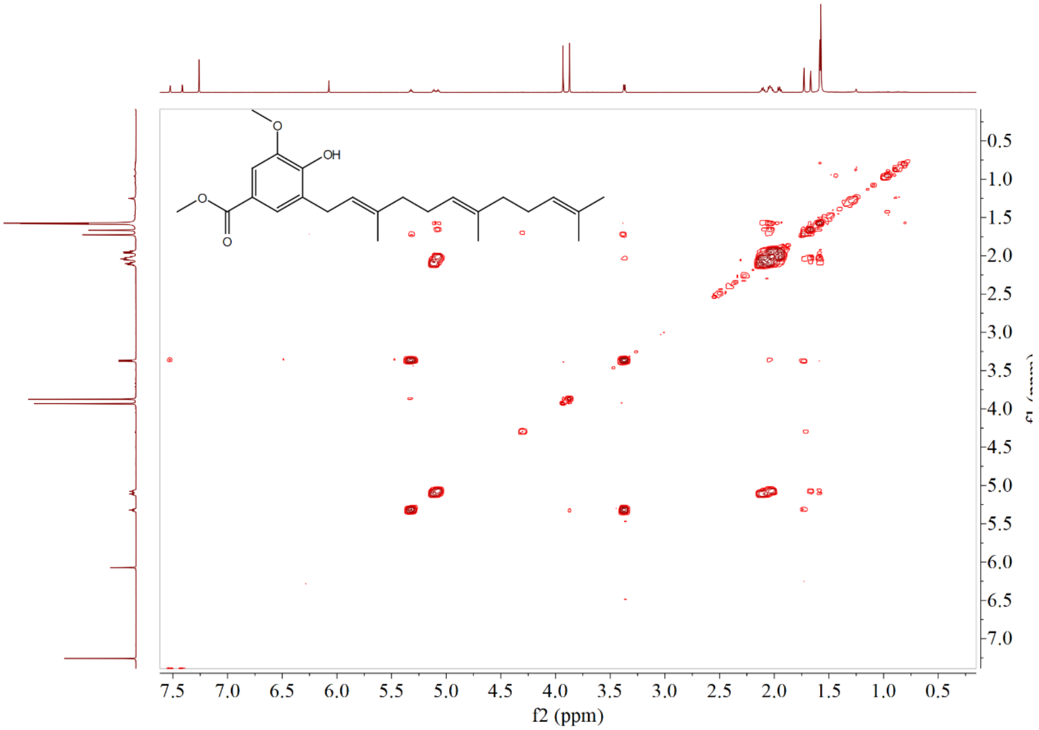

(f) ^{1}H-^{1}H COSY spectrum of compound 6 in CDCl$_3$

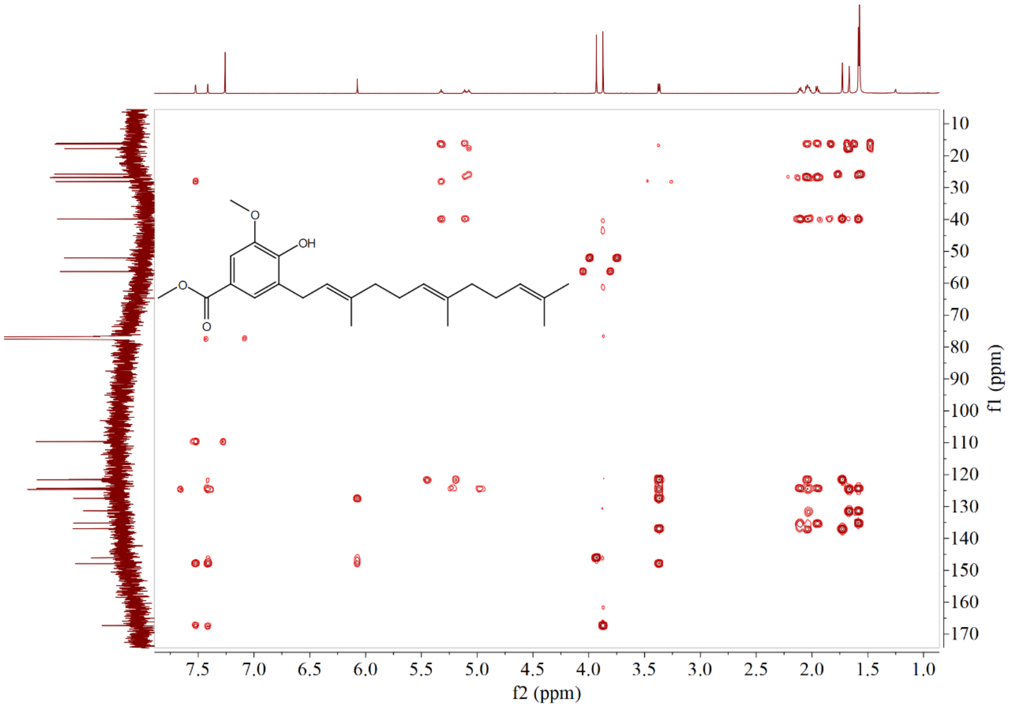

(g) HMBC spectrum of compound 6 in CDCl₃

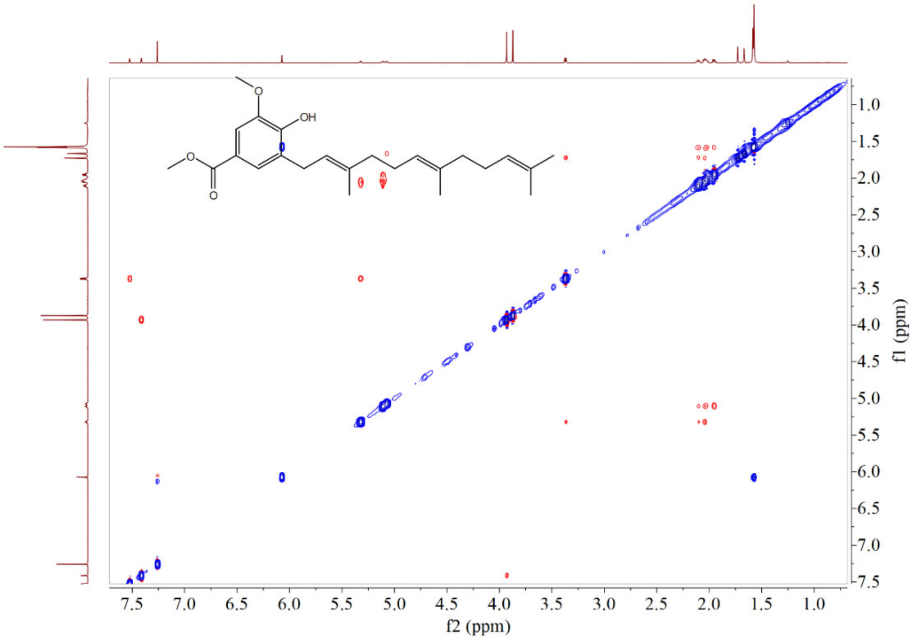

(h) NOESY spectrum of compound 6 in CDCl₃

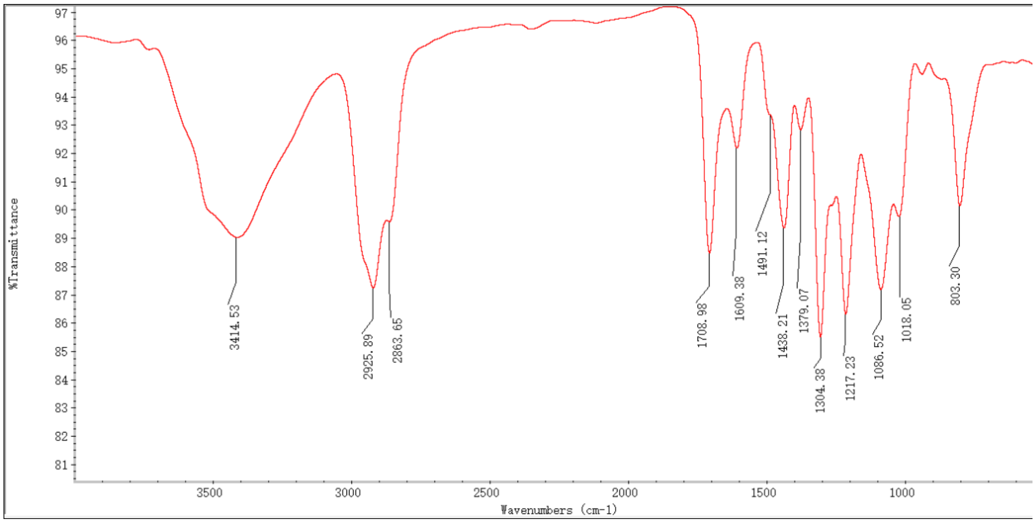

(i) FT-IR spectrum of compound 6

Figure S1. 1D, 2D NMR, MS and IR spectra of Compound 6.

Qualitative Analysis Report

**Data Filename**	ESIH202103218.d	**Sample Name**	A8-A8-3JHB1
**Sample ID**		**Position**	P1-E4
**Instrument Name**	Agilent G6520 Q-TOF	**Acq Method**	20160322_MS_ESIH_POS_1min.m
**Acquired Time**	6/30/2021 16:09:54	**IRM Calibration Status**	Success
**DA Method**	small molecular data analysis method.m	**Comment**	ESIH by zhuzhenyun

User Spectra

Fragmentor Voltage	Collision Energy	Ionization Mode
135	0	ESI

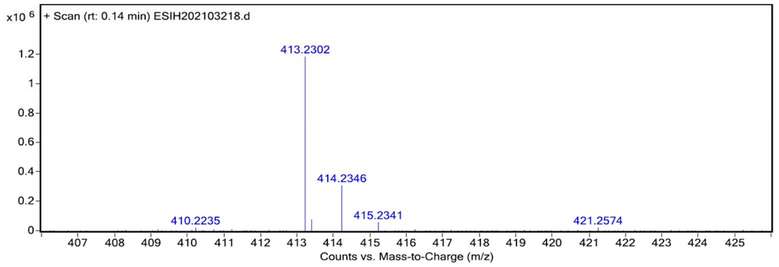

Formula Calculator Results

m/z	Calc m/z	Diff (mDa)	Diff (ppm)	Ion Formula	Ion
413.2302	413.2298	-0.32	-0.78	C23 H34 Na O5	(M+Na)+

--- End Of Report ---

(a) HRESIMS spectrum of compound 7

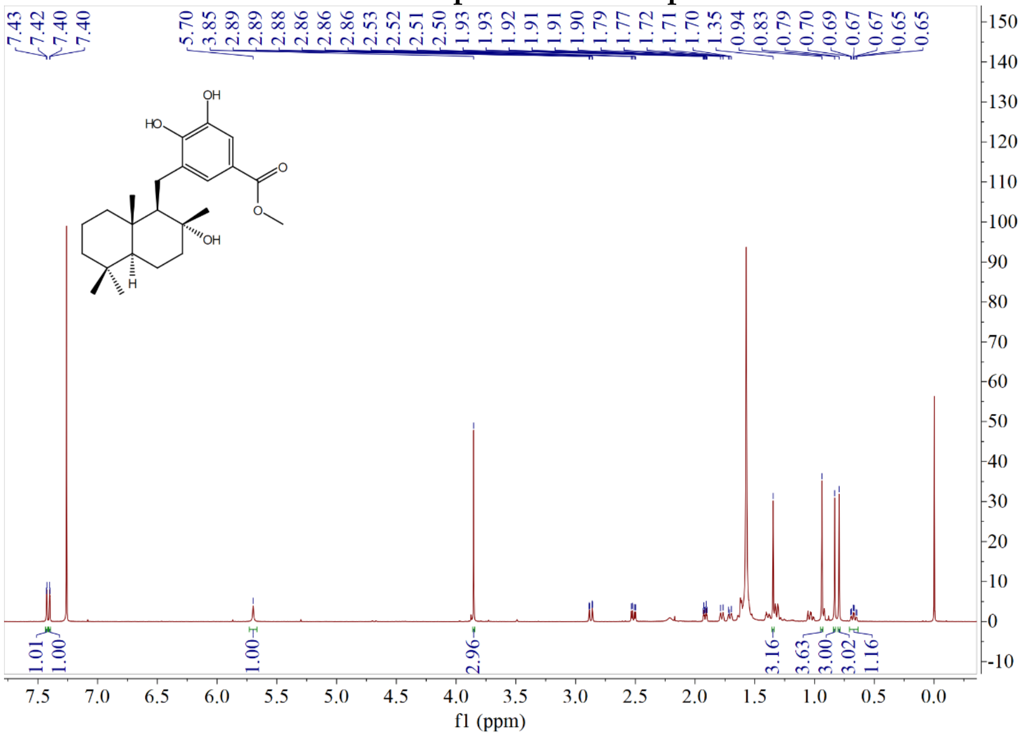

(b) ^{1}H NMR spectrum of compound 7 in $CDCl_3$

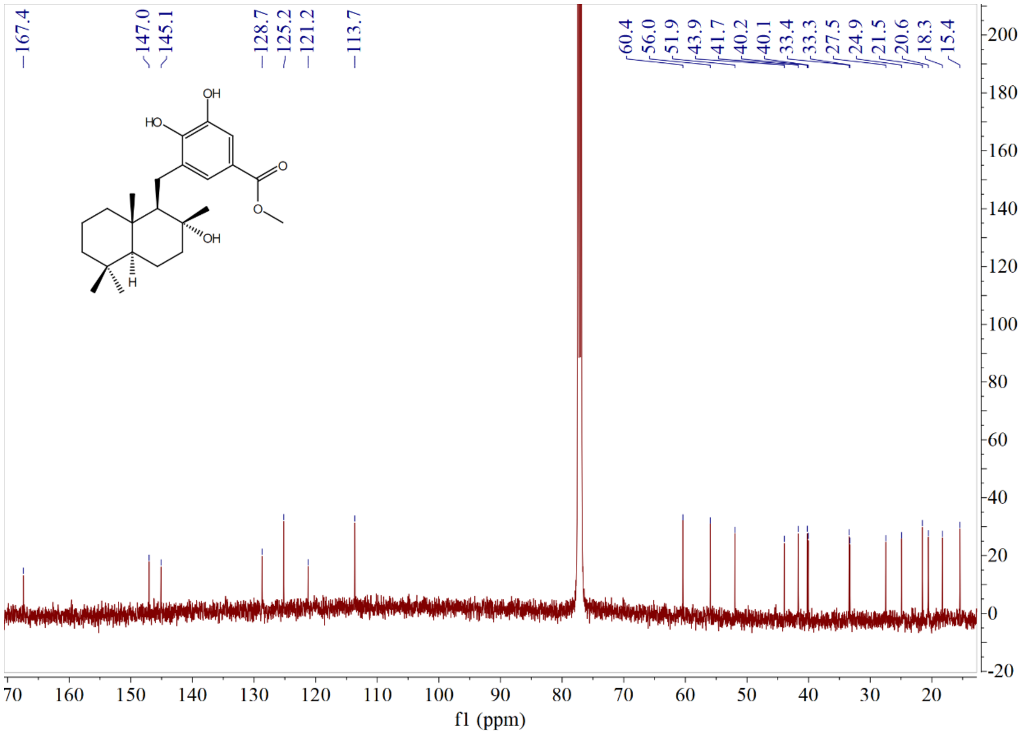

(c) ^{13}C NMR spectrum of compound 7 in $CDCl_3$

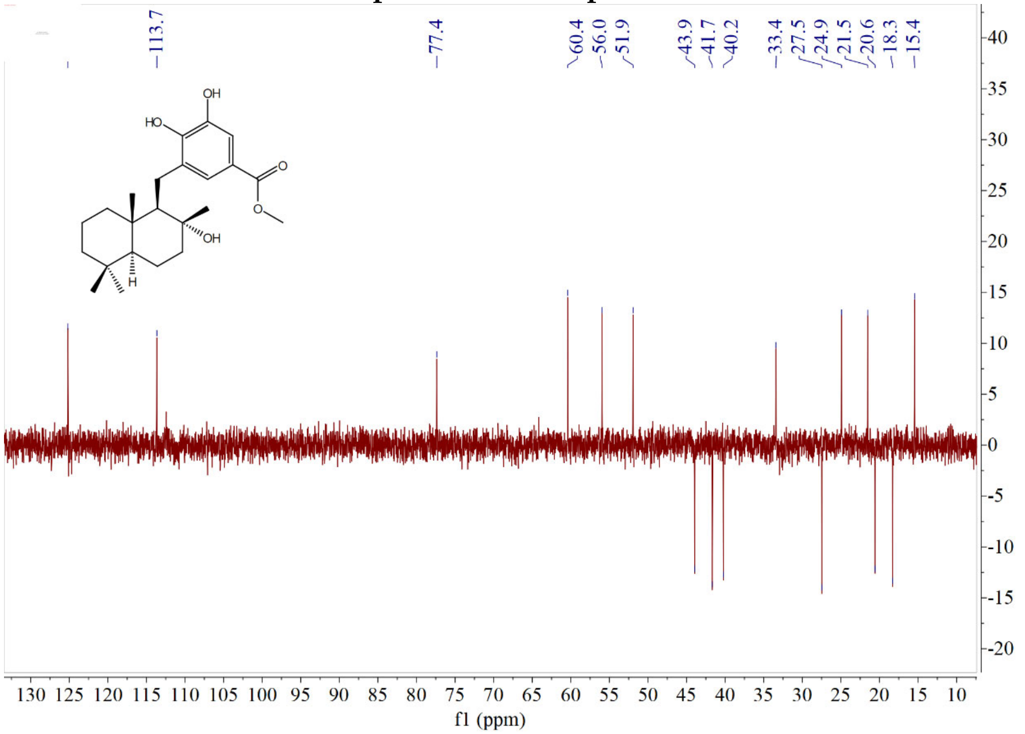

(d) DEPT spectrum of compound 7 in $CDCl_3$

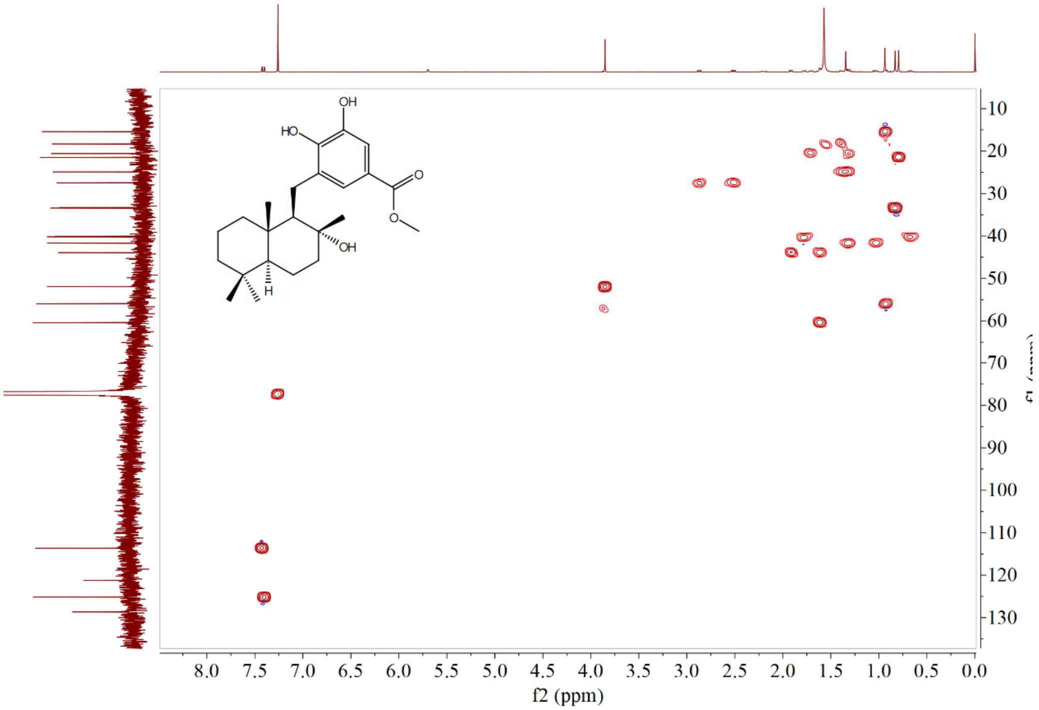

(e) HSQC spectrum of compound 7 in $CDCl_3$

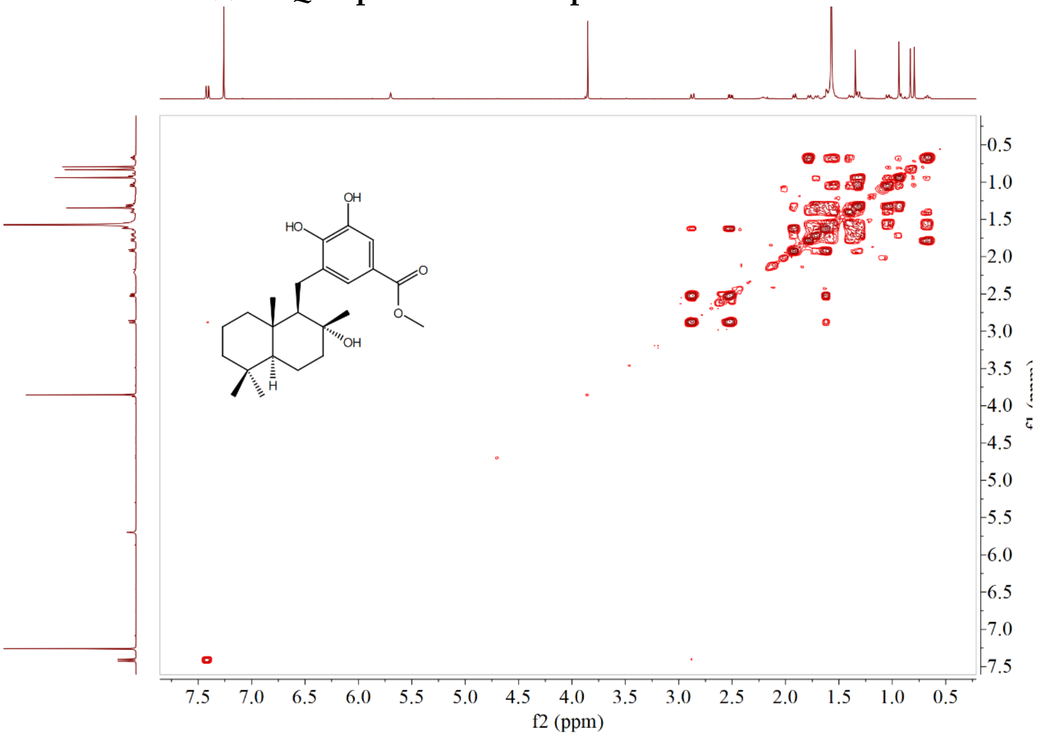

(f) ^{1}H-^{1}H COSY spectrum of compound 7 in $CDCl_3$

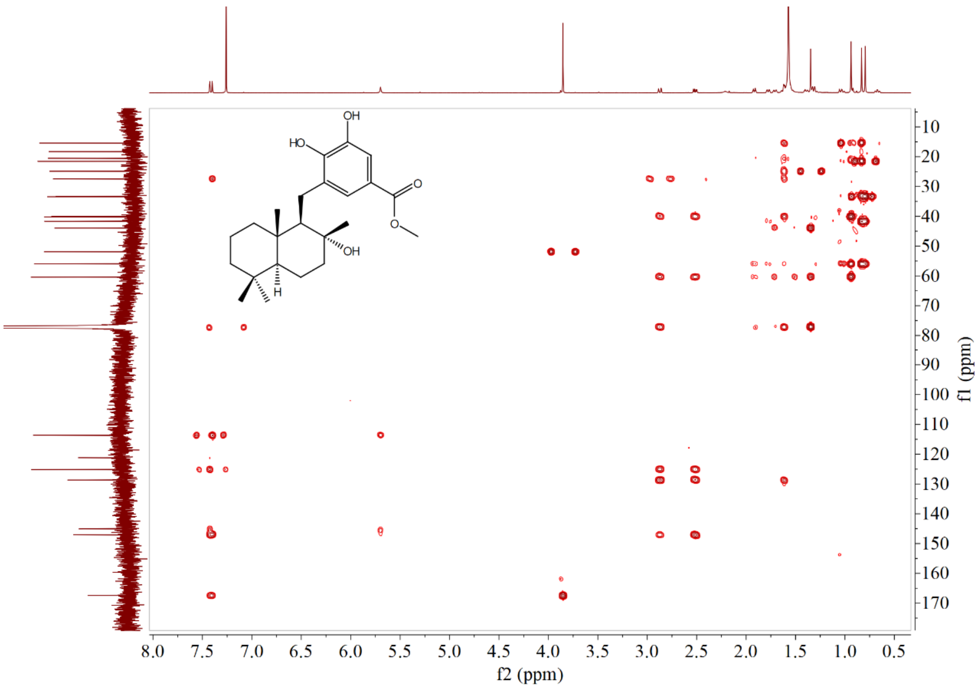

(g) HMBC spectrum of compound 7 in $CDCl_3$

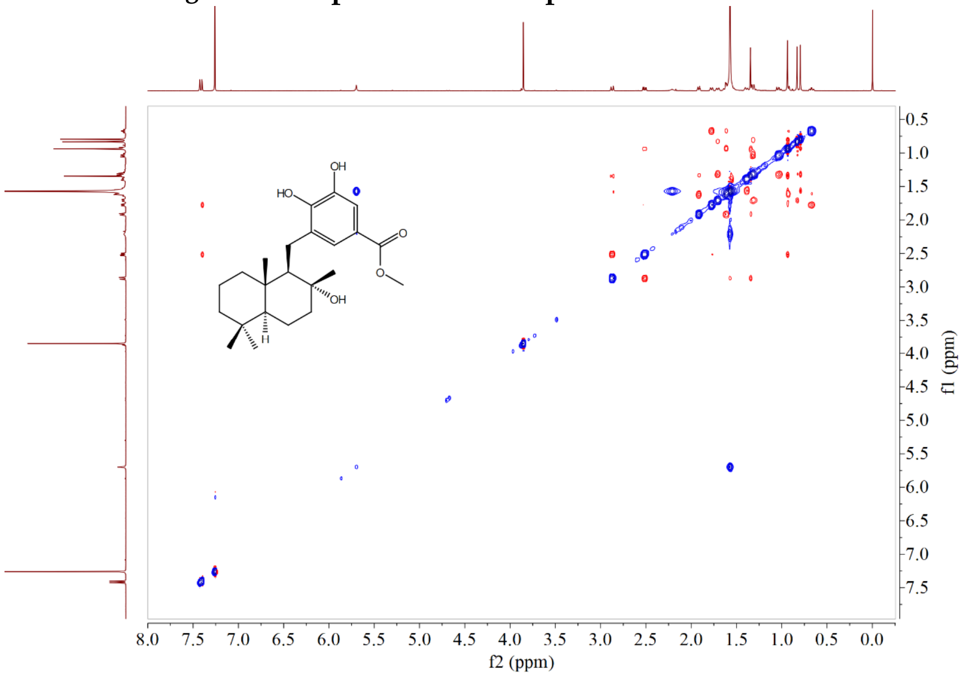

(h) NOESY spectrum of compound 7 in $CDCl_3$

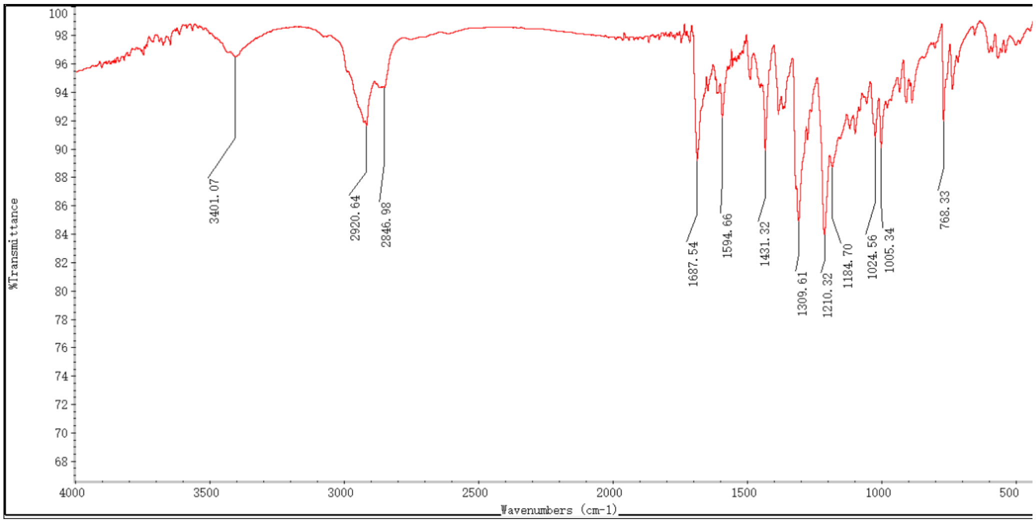

(i) FT-IR spectrum of compound 7

Figure S2 1D, 2D NMR, MS and IR spectra of Compound 7

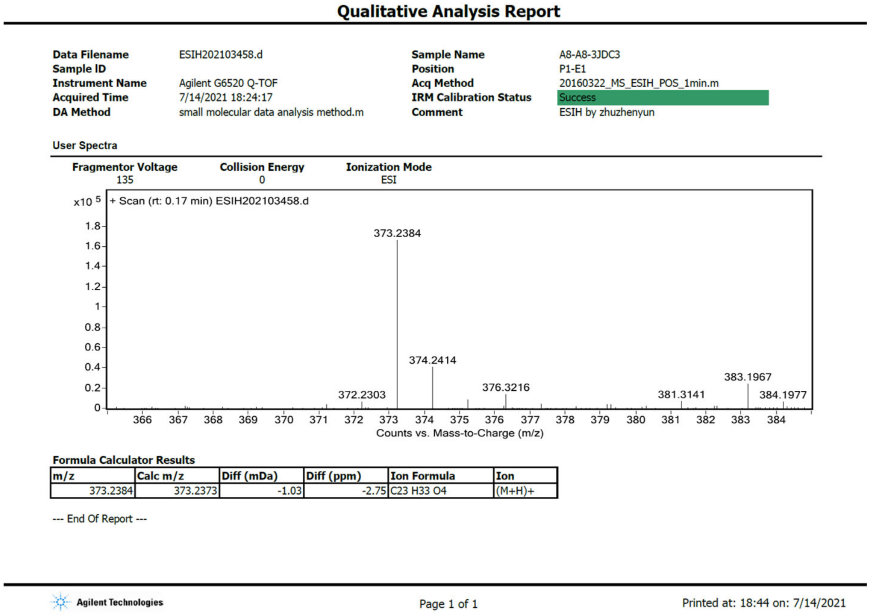
Qualitative Analysis Report

Data Filename	ESIH202103458.d	Sample Name	A8-A8-3JDC3
Sample ID		Position	P1-E1
Instrument Name	Agilent G6520 Q-TOF	Acq Method	20160322_MS_ESIH_POS_1min.m
Acquired Time	7/14/2021 18:24:17	IRM Calibration Status	Success
DA Method	small molecular data analysis method.m	Comment	ESIH by zhuzhenyun

User Spectra

Fragmentor Voltage	Collision Energy	Ionization Mode
135	0	ESI

Formula Calculator Results

m/z	Calc m/z	Diff (mDa)	Diff (ppm)	Ion Formula	Ion
373.2384	373.2373	-1.03	-2.75	C23 H33 O4	(M+H)+

--- End Of Report ---

Agilent Technologies Page 1 of 1 Printed at: 18:44 on: 7/14/2021

(a) HRESIMS spectrum of compound 8

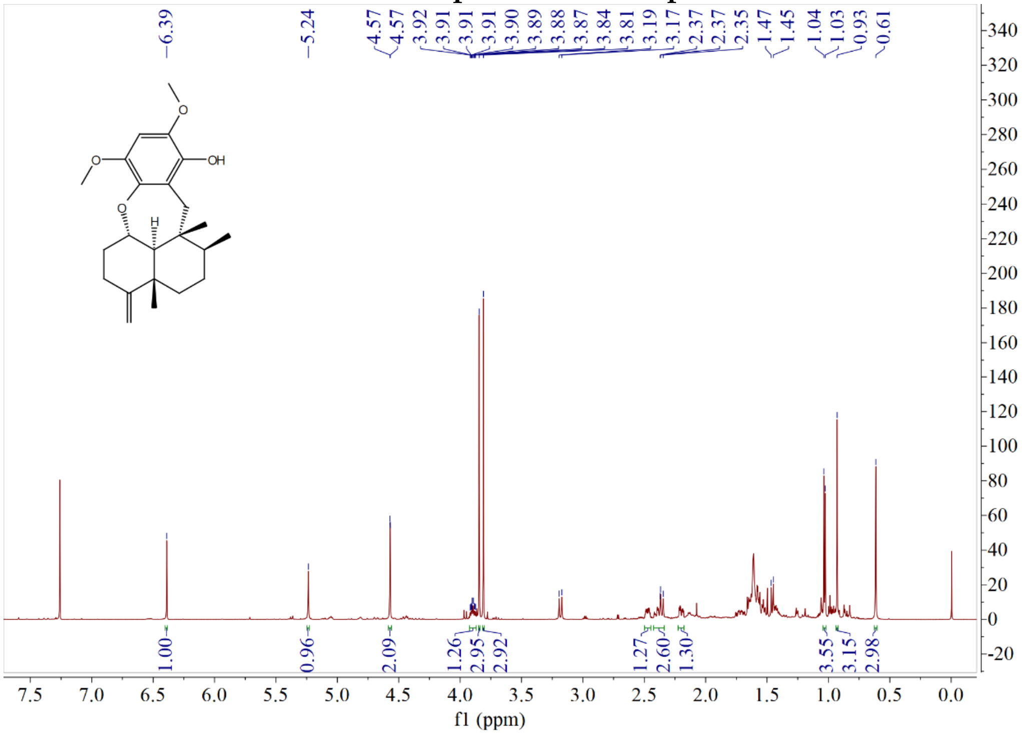

(b) ^{1}H NMR spectrum of compound 8 in $CDCl_3$

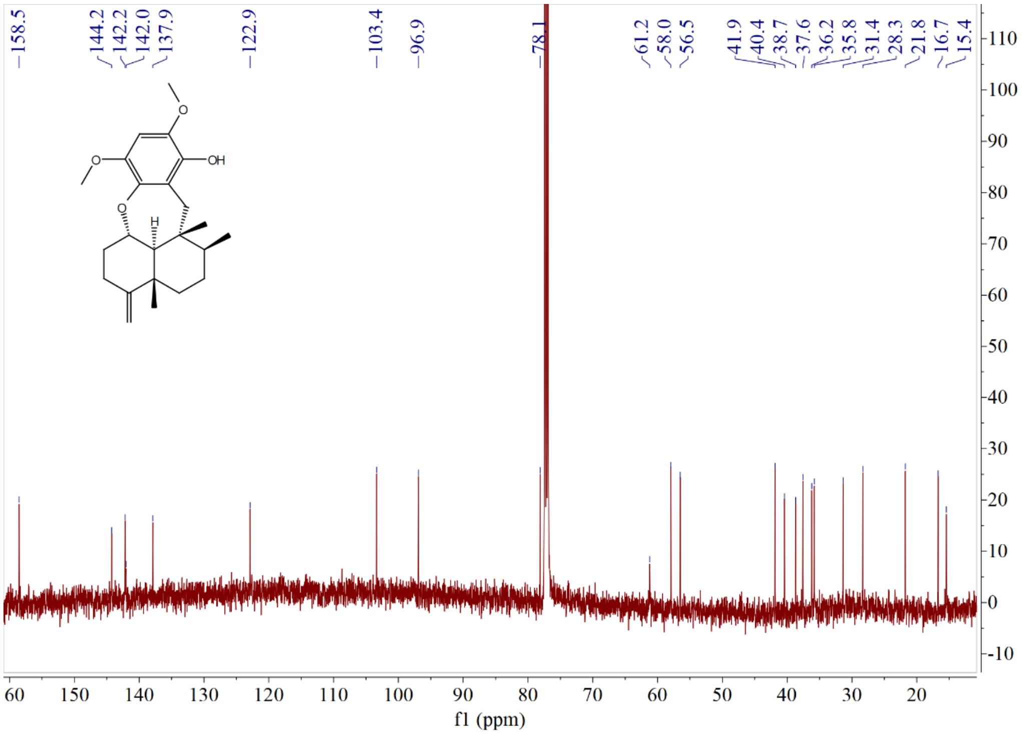

(c) **^{13}C NMR spectrum of compound 8 in $CDCl_3$**

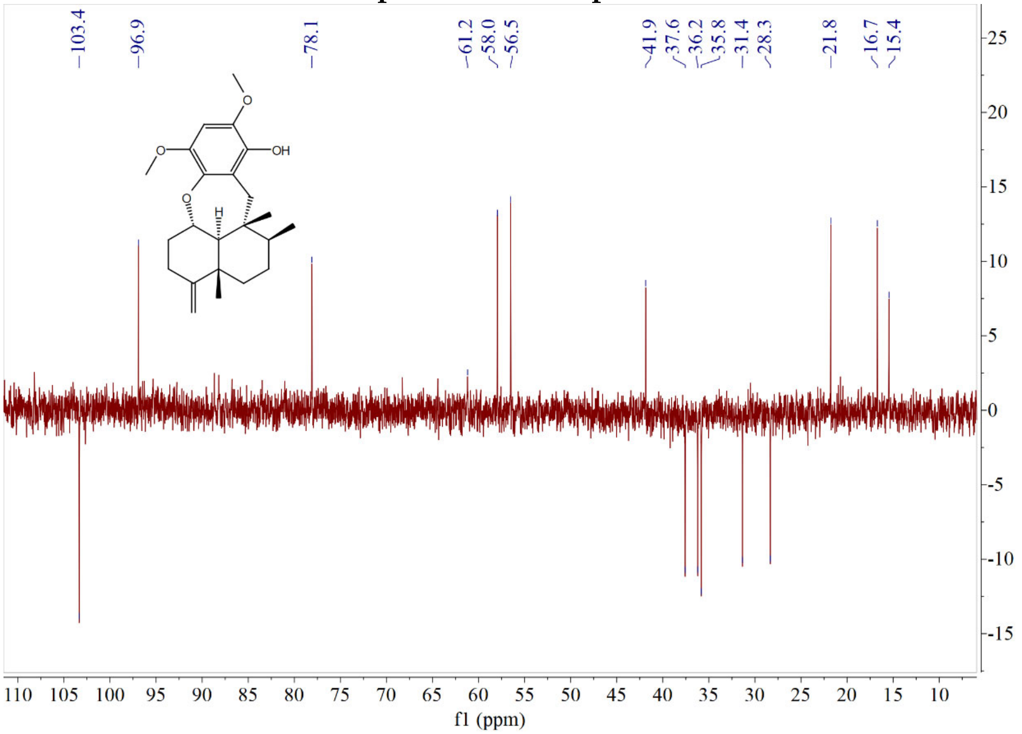

(d) **DEPT spectrum of compound 8 in $CDCl_3$**

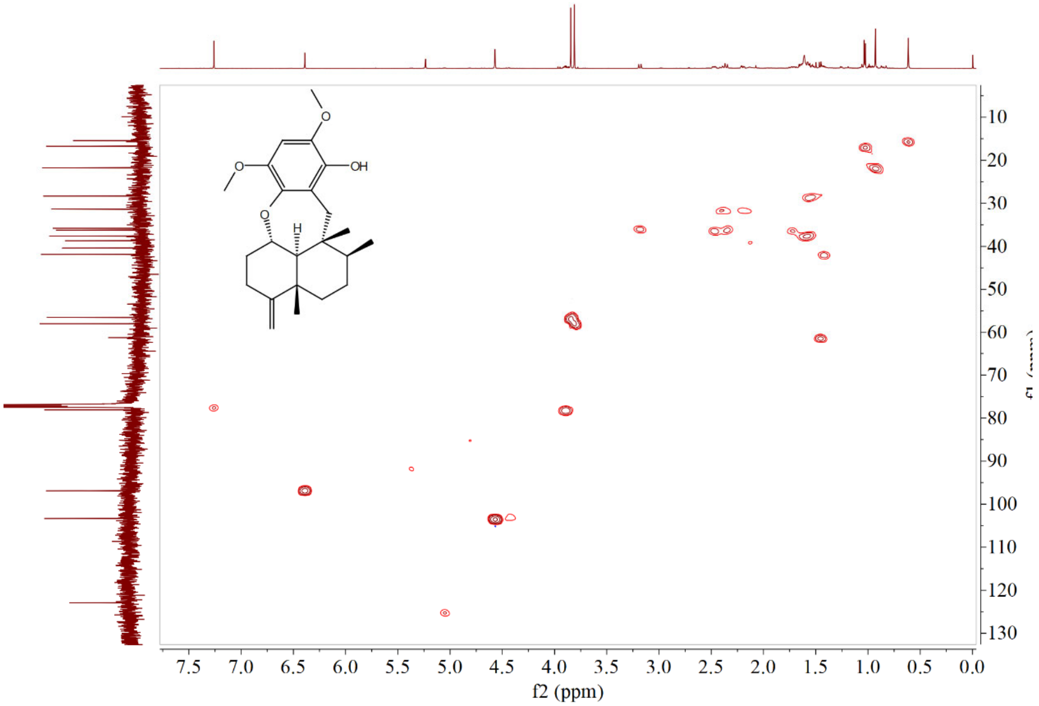

(e) HSQC spectrum of compound 8 in $CDCl_3$

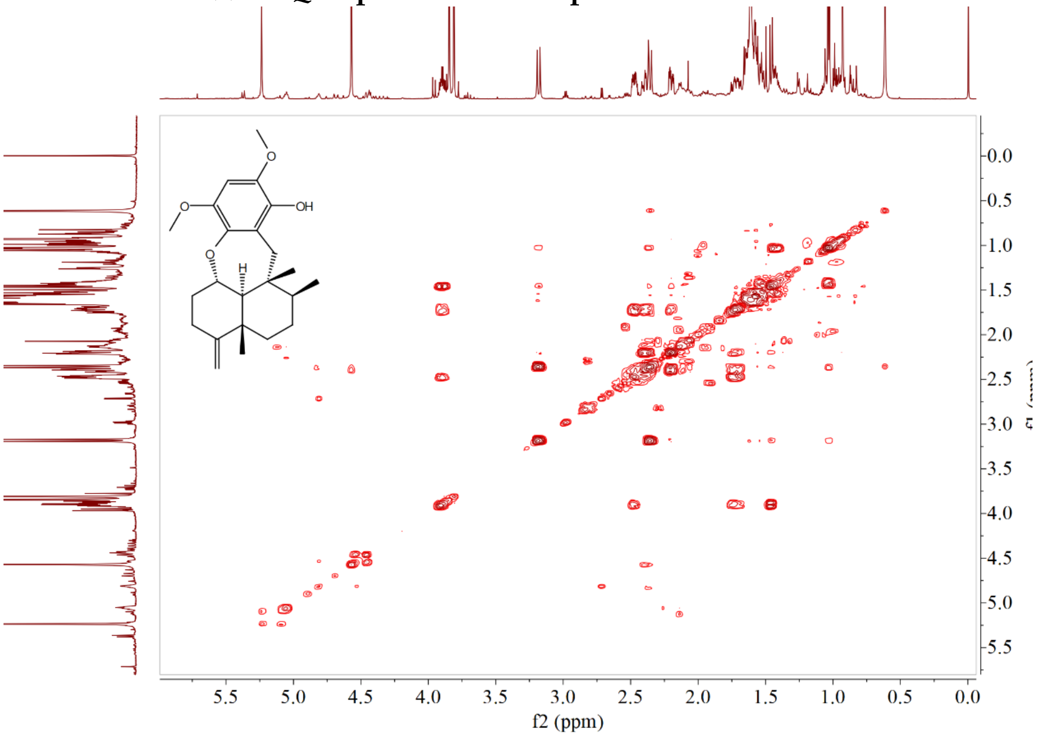

(f) ^1H-^1H COSY spectrum of compound 8 in $CDCl_3$

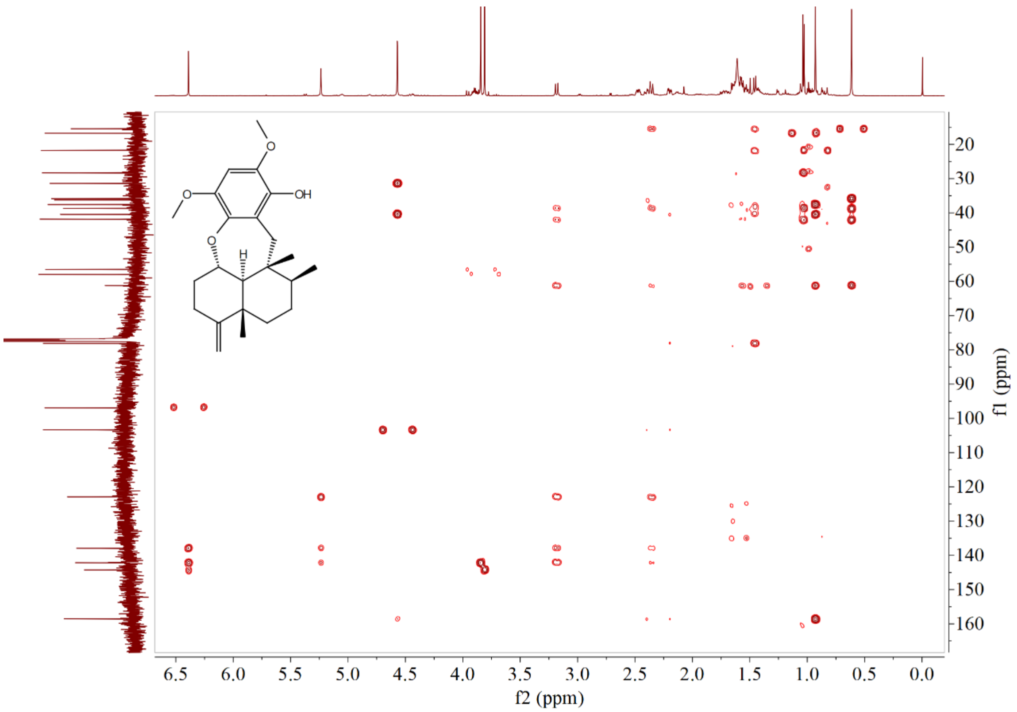

(g) HMBC spectrum of compound 8 in $CDCl_3$

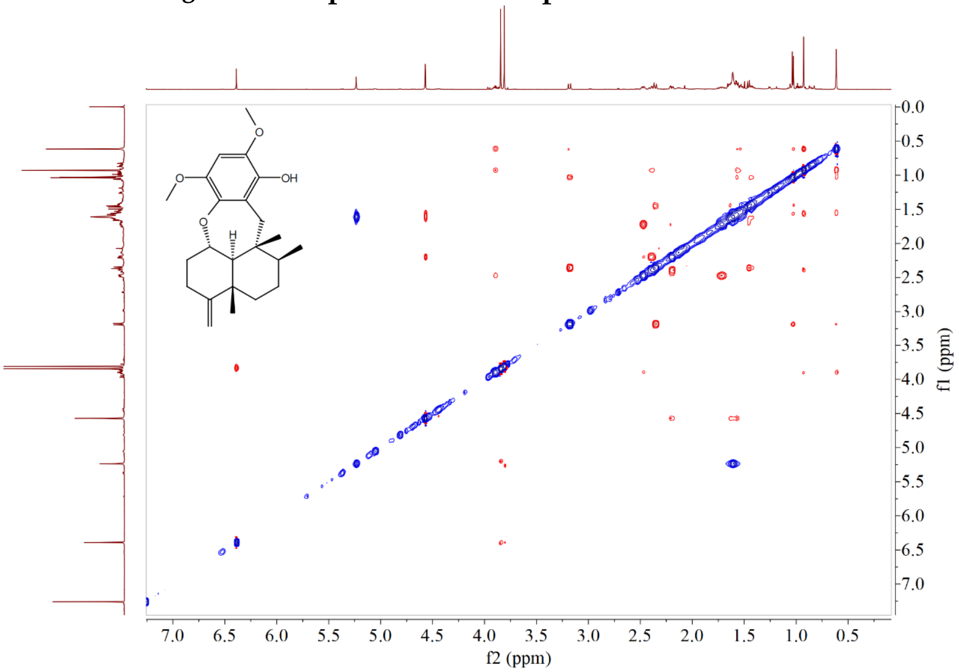

(h) NOESY spectrum of compound 8 in $CDCl_3$

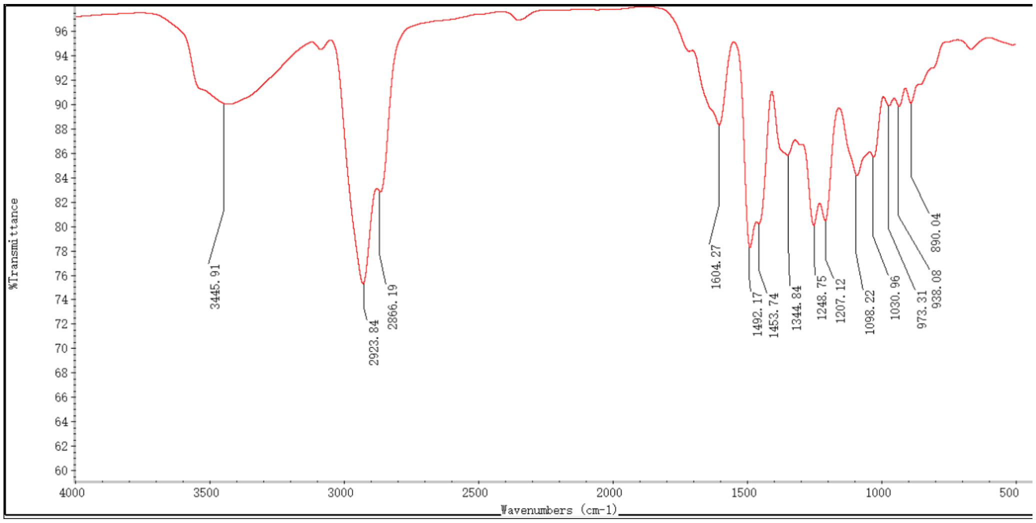

(i) FT-IR spectrum of compound 8

Figure S3 1D, 2D NMR, MS and IR spectra of Compound 8

D:\data\2021\EI202101307_A8-A8-2JGC4 -c1 6/15/2021 8:54:37 AM

EI202101307_A8-A8-2JGC4 -c1#7 RT: 1.24
T: + c EI Full ms [49.50-800.50]
m/z= 48-803

m/z	Intensity	Relative	Theo. Mass	Delta (mmu)	RDB equiv.	Composition
205.0858	396046.0	11.86	205.0859	-0.15	6.5	$C_{12}H_{13}O_3$
217.0858	228612.0	6.84	217.0859	-0.16	7.5	$C_{13}H_{13}O_3$
218.0939	311565.0	9.33	218.0937	0.11	7.0	$C_{13}H_{14}O_3$
219.1013	236693.0	7.09	219.1016	-0.30	6.5	$C_{13}H_{15}O_3$
220.1097	1258839.0	37.69	220.1094	0.35	6.0	$C_{13}H_{16}O_3$
231.1013	262170.0	7.85	231.1016	-0.27	7.5	$C_{14}H_{15}O_3$
232.1090	509735.0	15.26	232.1094	-0.40	7.0	$C_{14}H_{16}O_3$
233.1167	465511.0	13.94	233.1172	-0.56	6.5	$C_{14}H_{17}O_3$
356.2346	1504553.0	45.04	356.2346	0.00	8.0	$C_{23}H_{32}O_3$

(a) HREIMS spectrum of compound 9

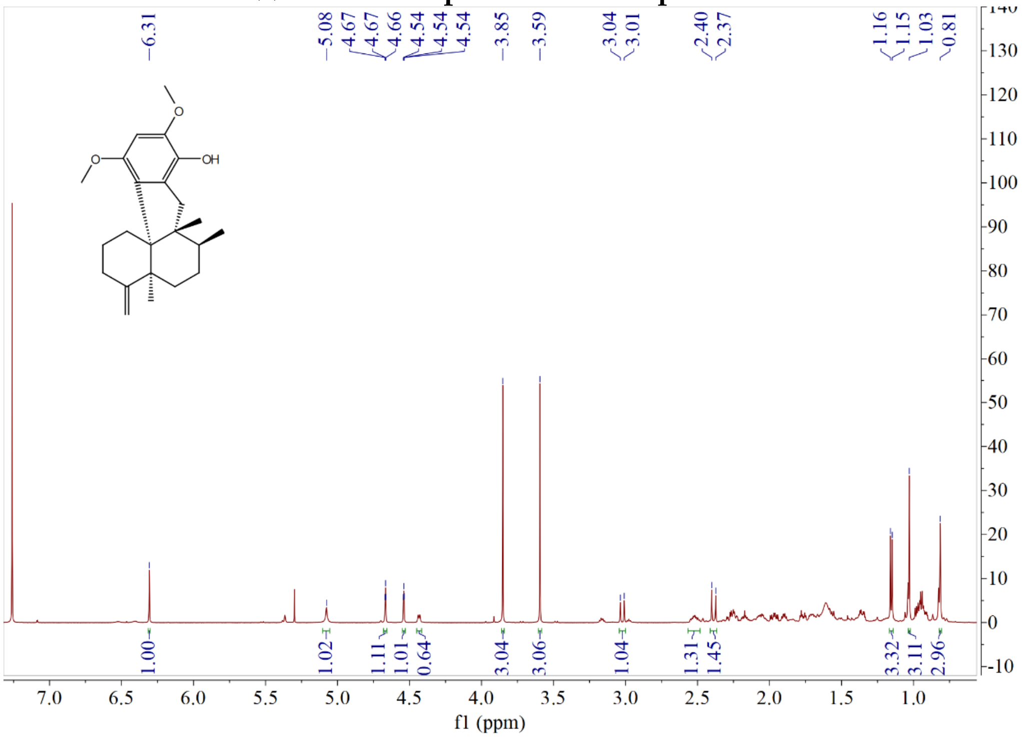

(b) ^{1}H NMR spectrum of compound 9 in $CDCl_3$

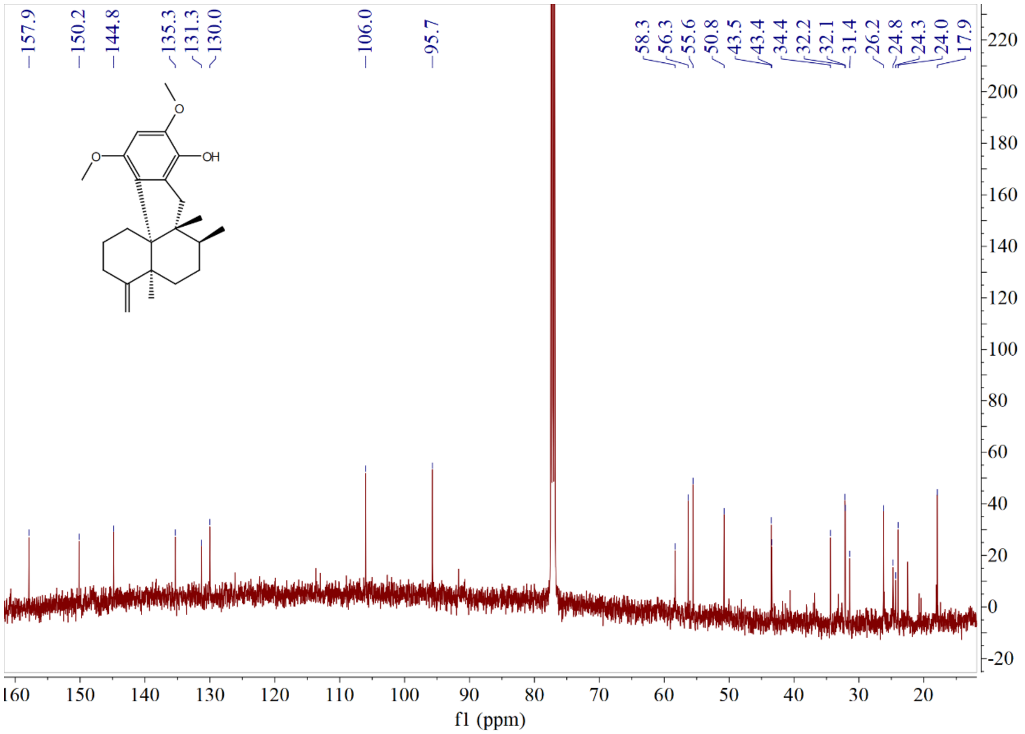

(c) ^{13}C NMR spectrum of compound 9 in $CDCl_3$

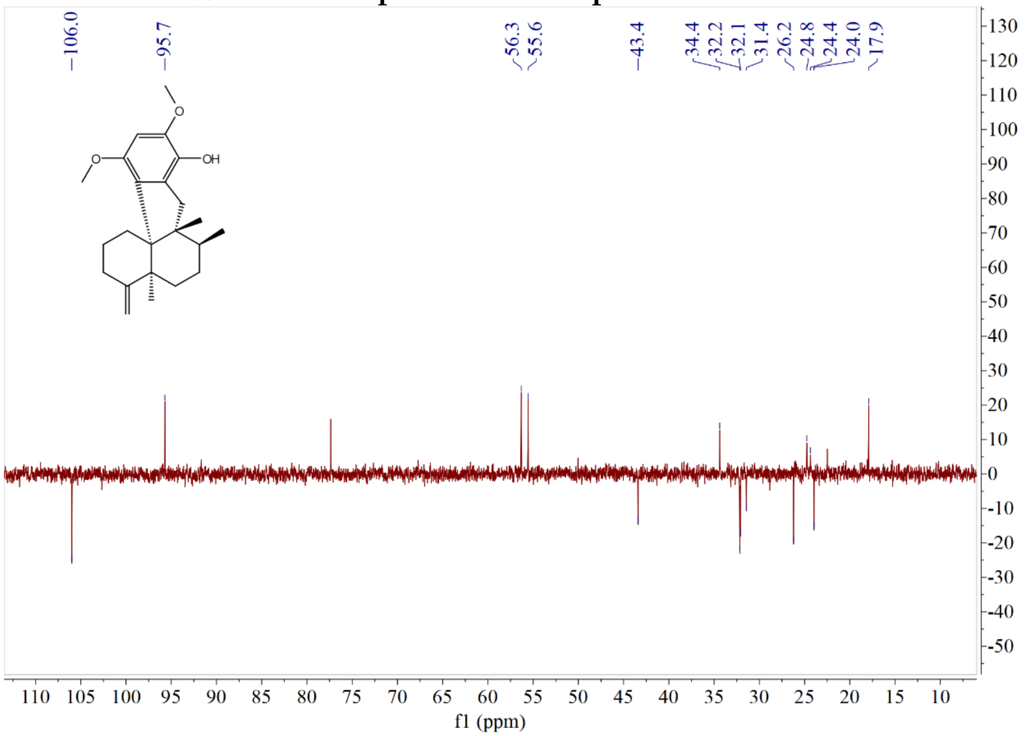

(d) DEPT spectrum of compound 9 in $CDCl_3$

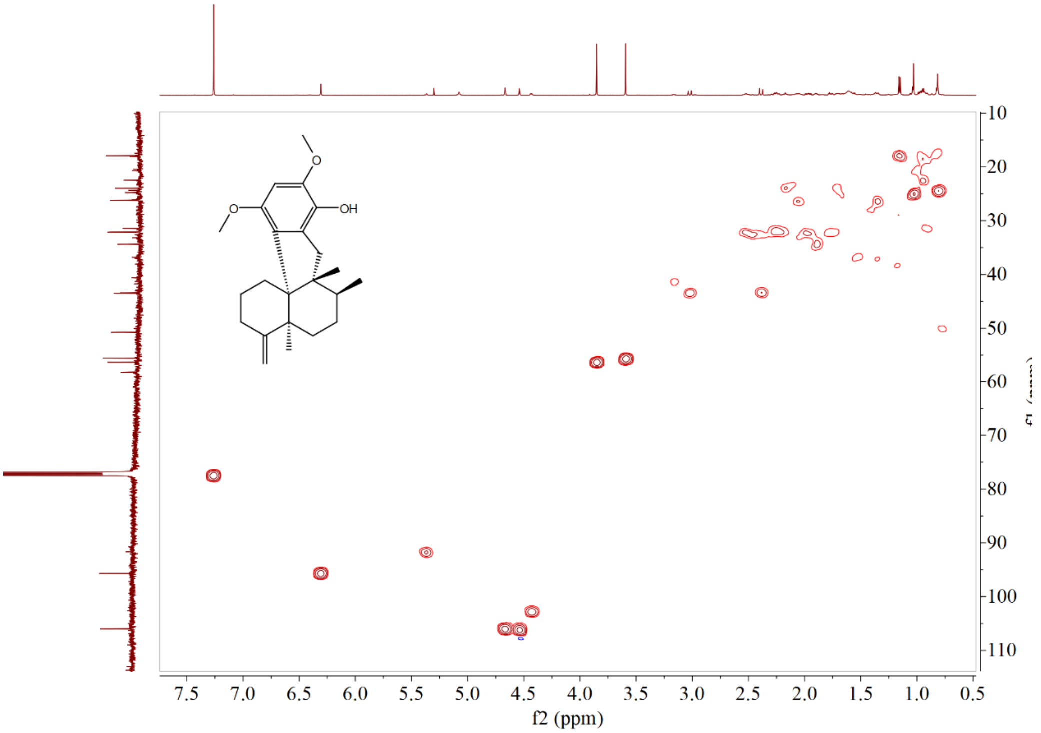

(e) HSQC spectrum of compound 9 in $CDCl_3$

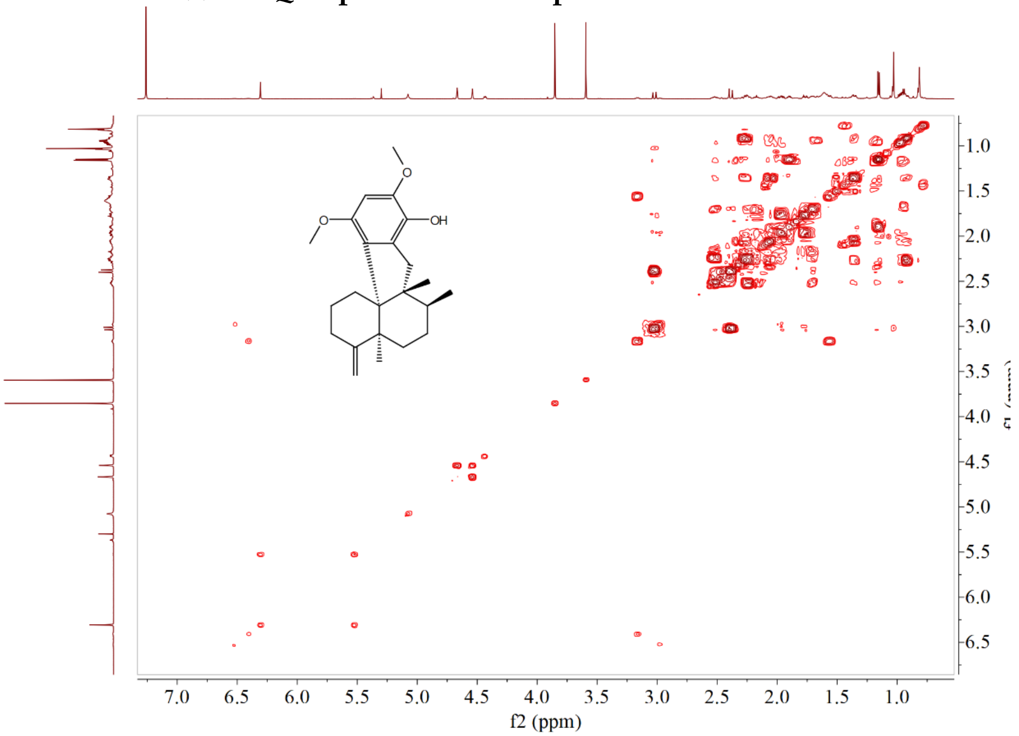

(f) ^{1}H-^{1}H COSY spectrum of compound 9 in $CDCl_3$

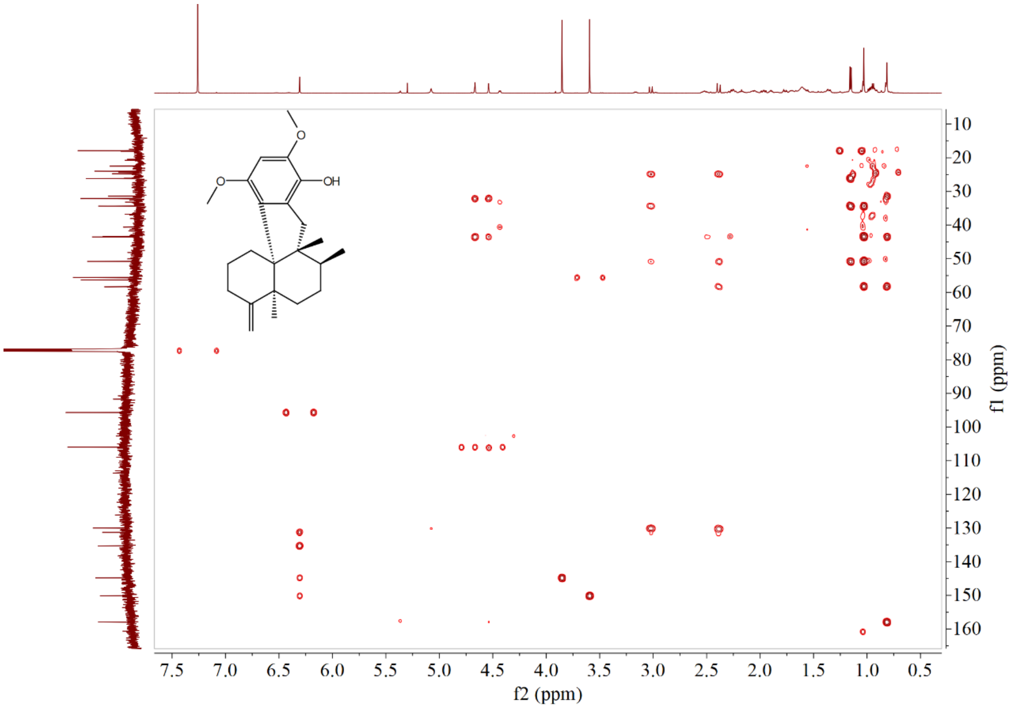

(g) HMBC spectrum of compound 9 in $CDCl_3$

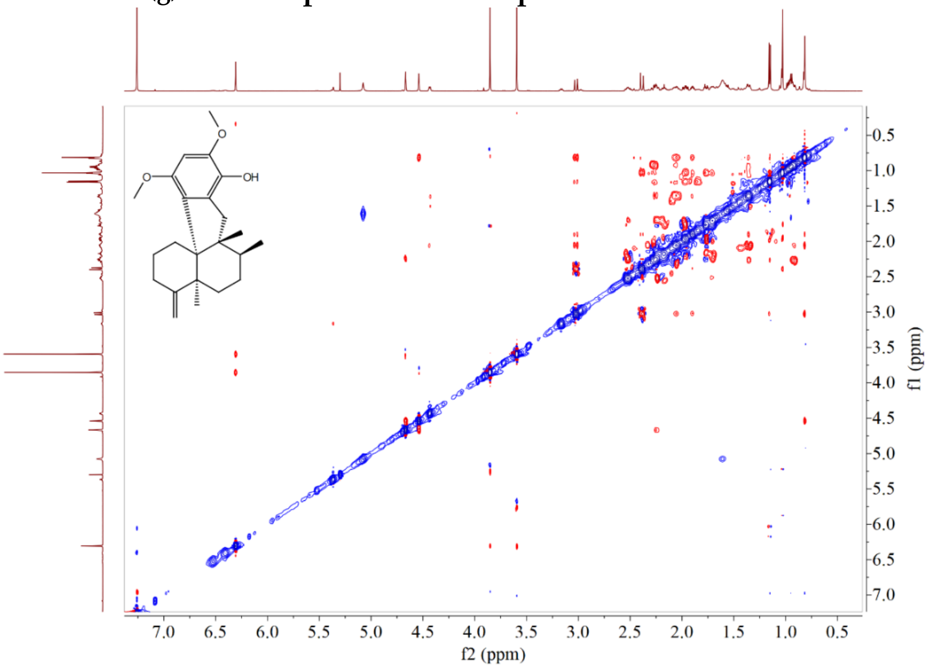

(h) NOESY spectrum of compound 9 in $CDCl_3$

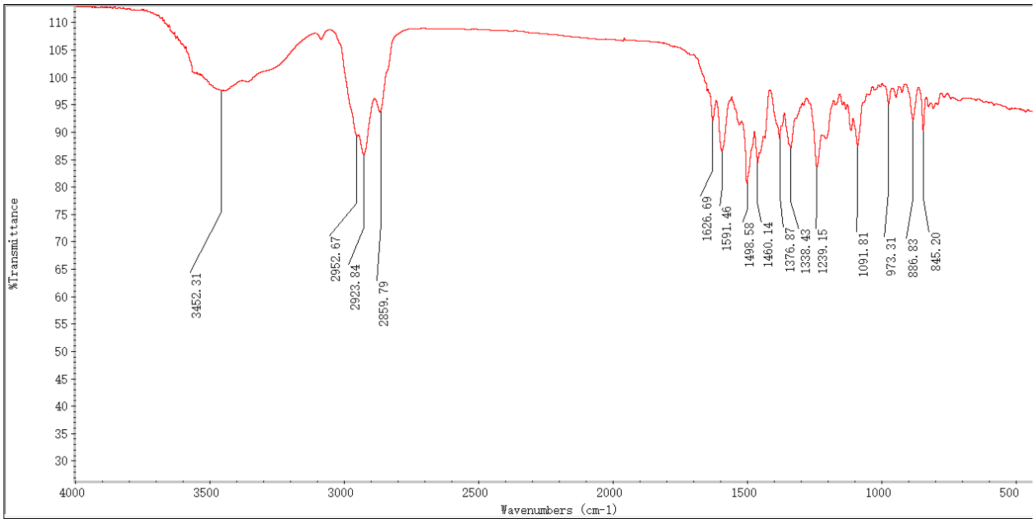

(i) FT-IR spectrum of compound 9

Figure S4 1D, 2D NMR, MS and IR spectra of Compound 9

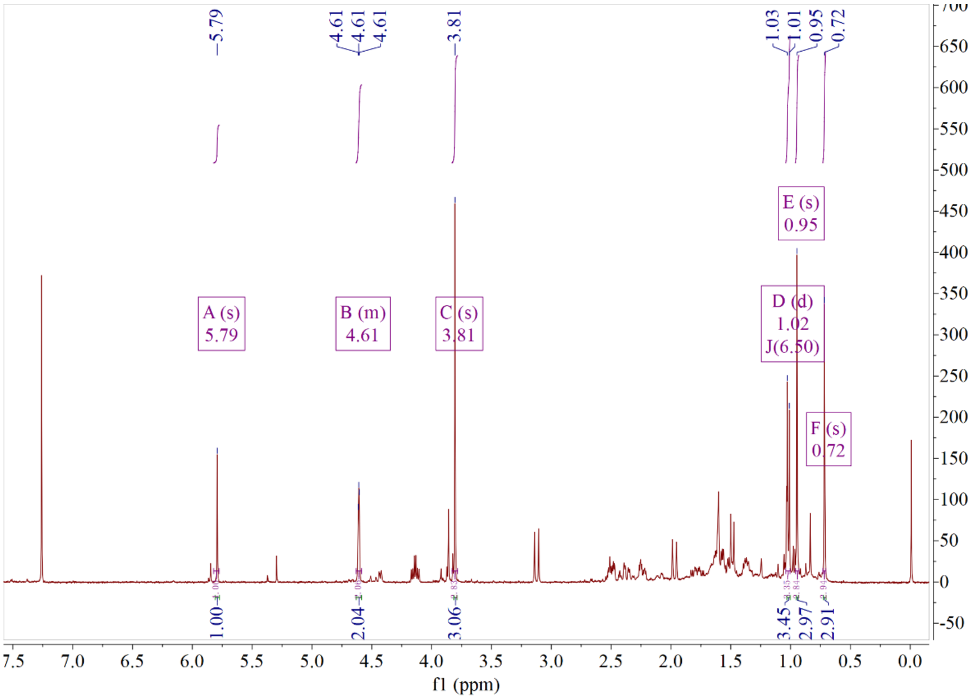

(a) ^{1}**H NMR spectrum of compound 12 in** $CDCl_3$

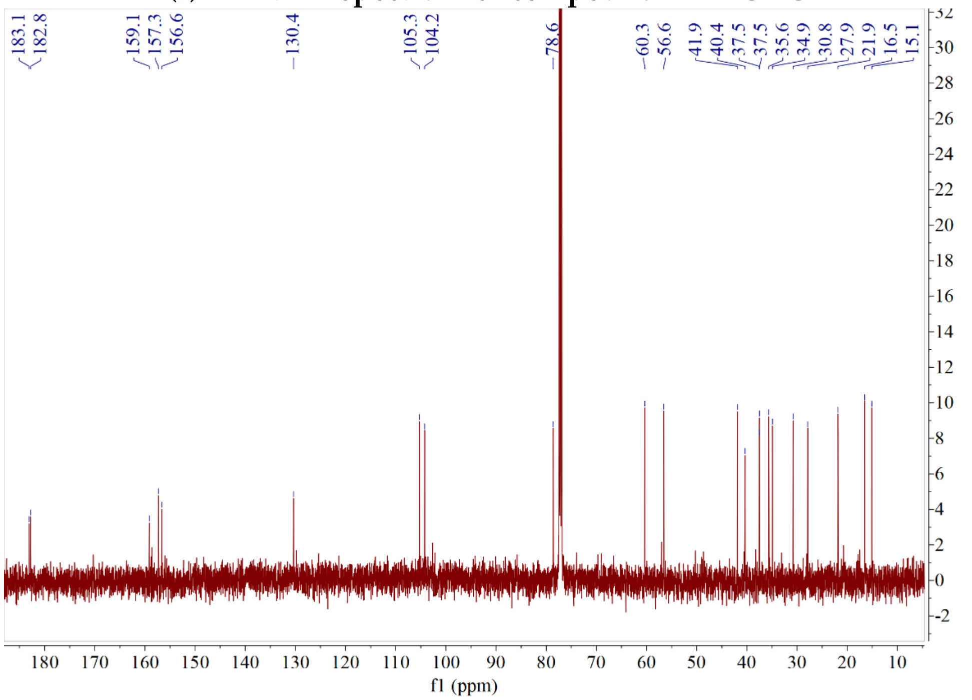

(b) ^{13}**C NMR spectrum of compound 12 in** $CDCl_3$

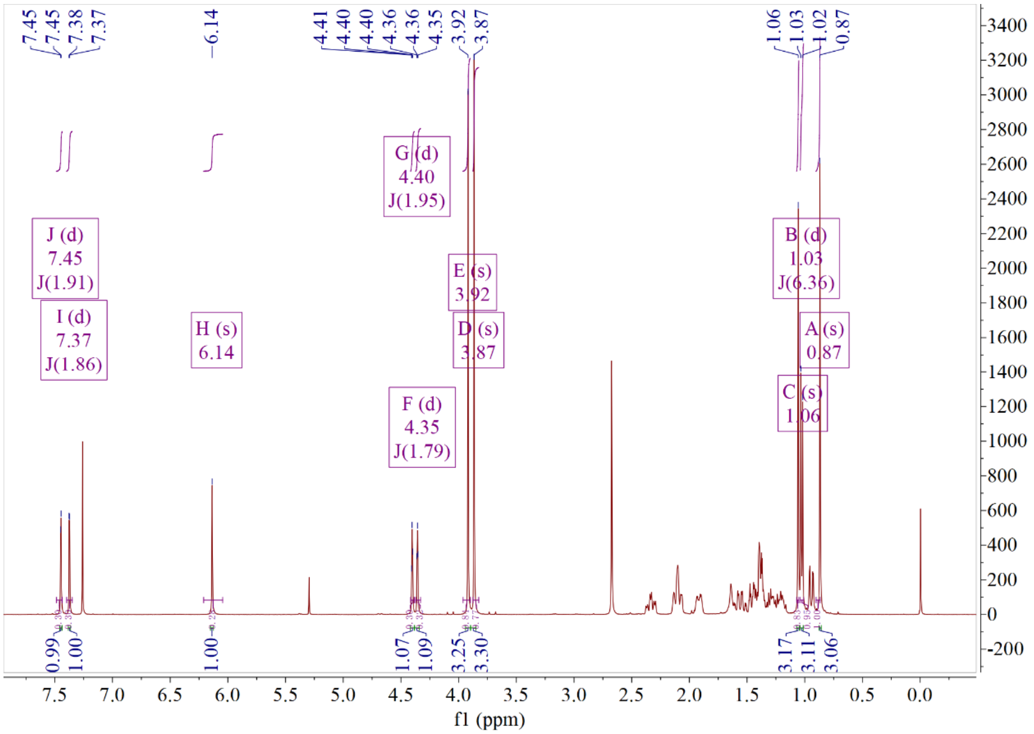

(c) ^{1}H NMR spectrum of compound 14 in $CDCl_3$

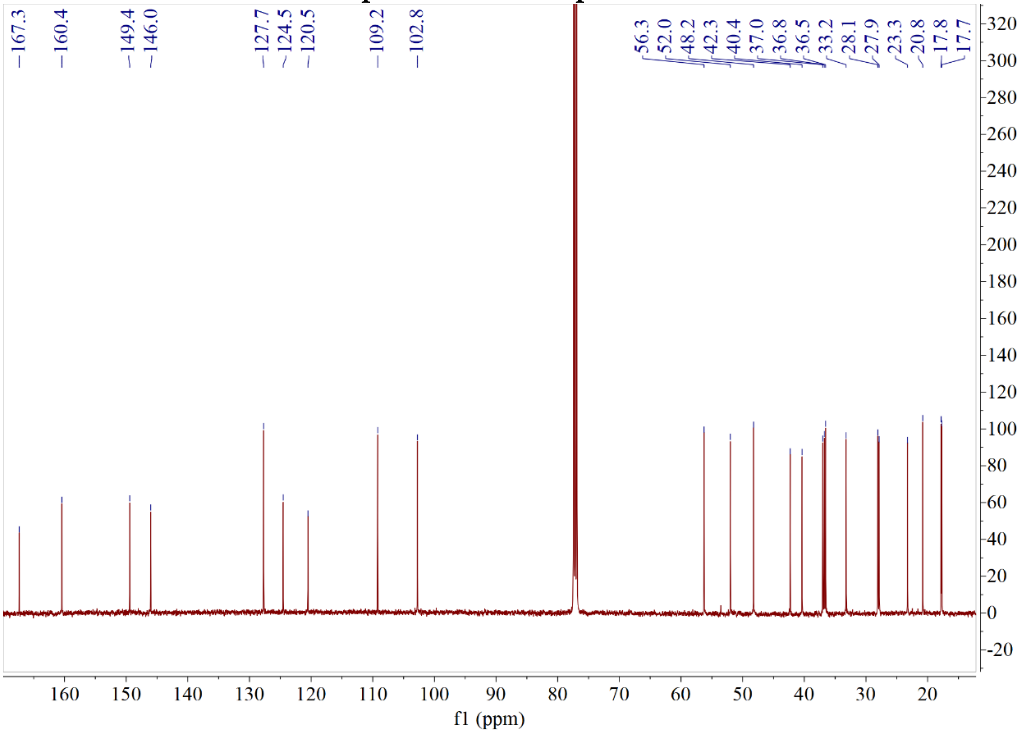

(d) ^{13}C NMR spectrum of compound 14 in $CDCl_3$

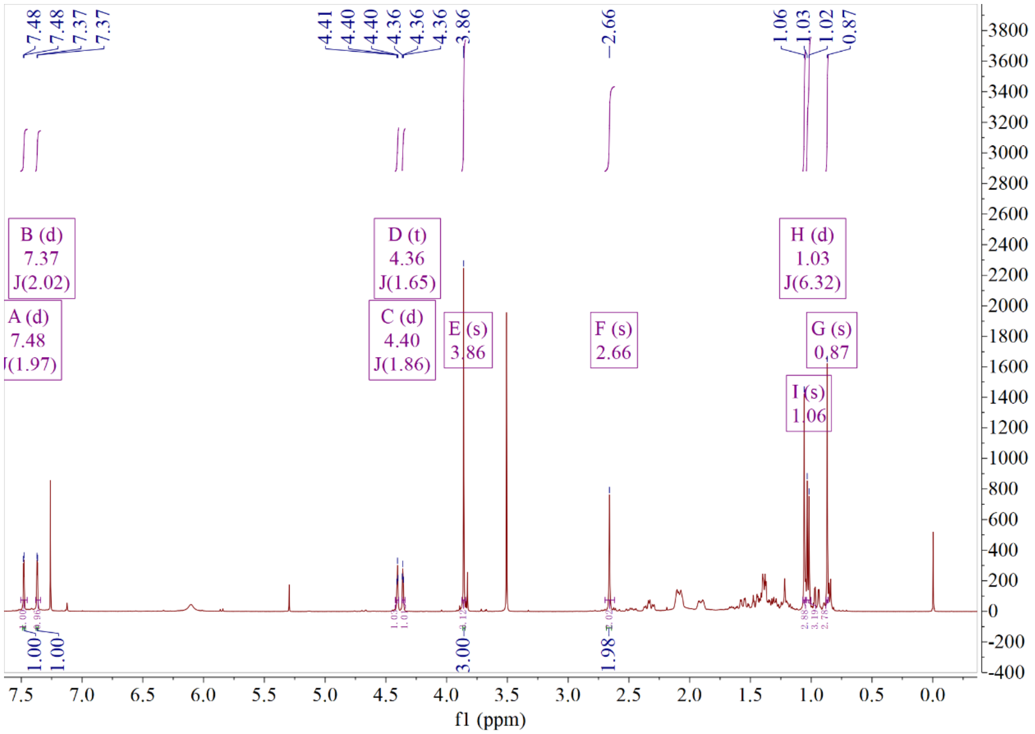

(e) ^{1}H NMR spectrum of compound 15 in $CDCl_3$

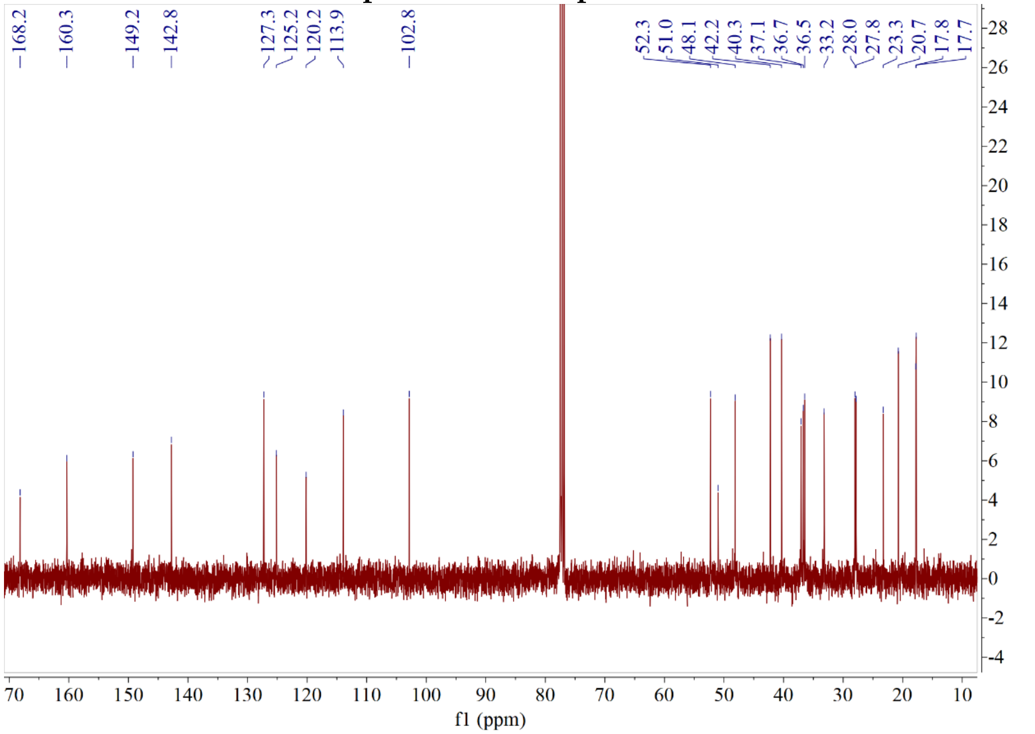

(f) ^{13}C NMR spectrum of compound 15 in $CDCl_3$

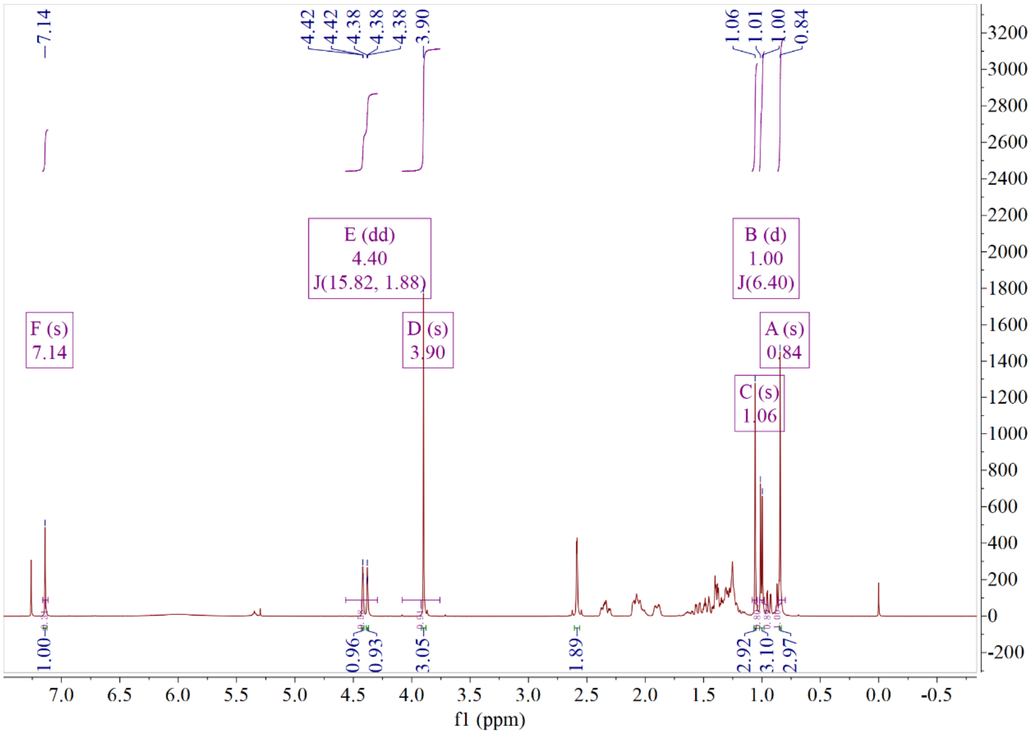

(g) ^{1}H NMR spectrum of compound 16 in CDCl$_3$

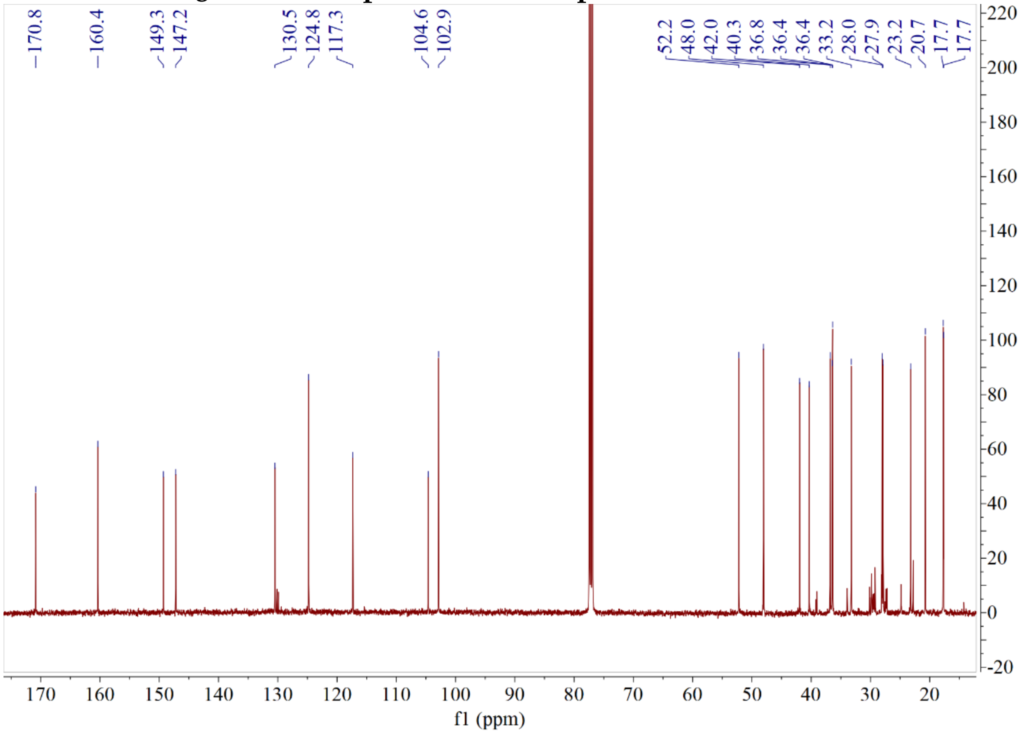

(h) ^{13}C NMR spectrum of compound 16 in CDCl$_3$

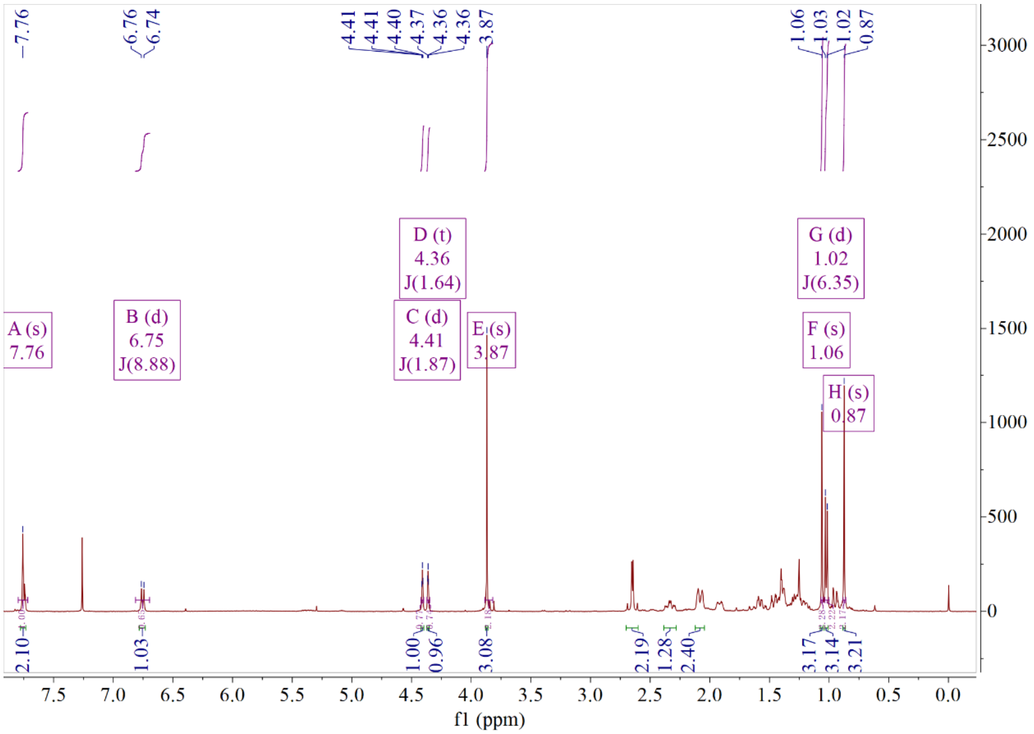

(i) ^{1}H NMR spectrum of compound 17 in $CDCl_3$

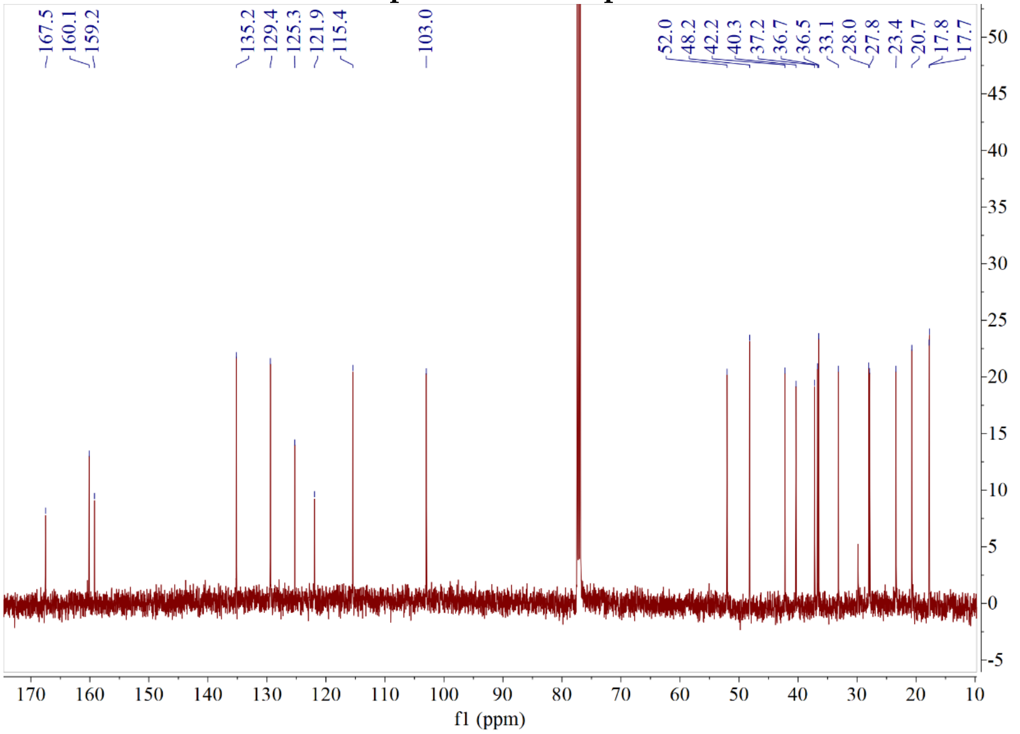

(j) ^{13}C NMR spectrum of compound 17 in $CDCl_3$

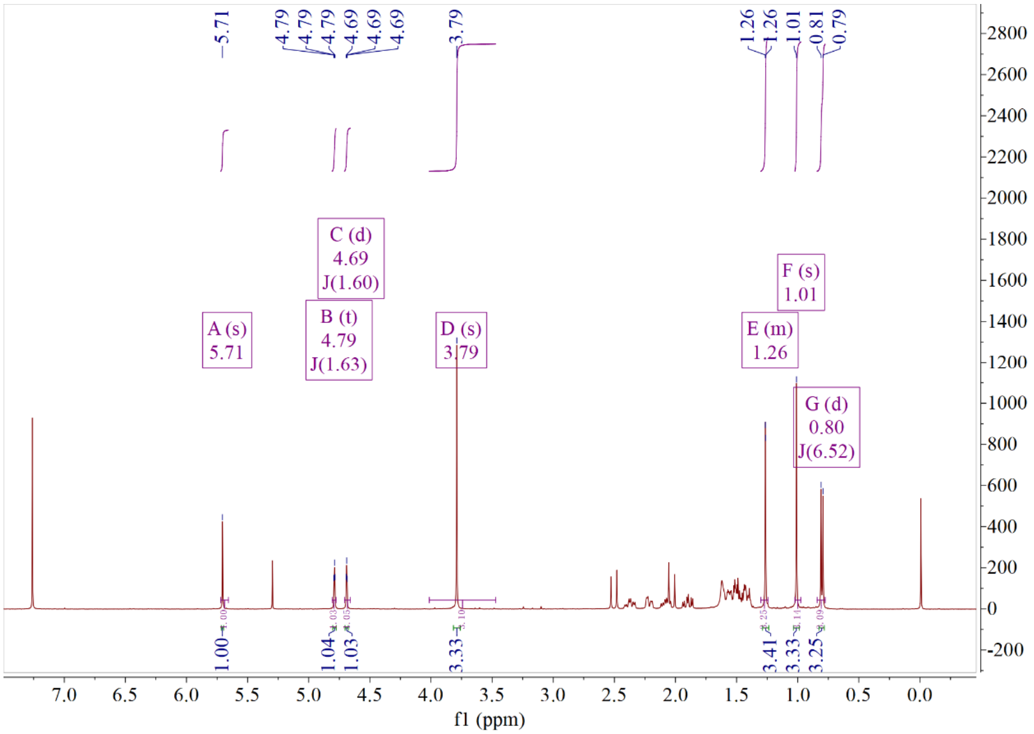

(k) ^{1}H NMR spectrum of compound 18 in $CDCl_3$

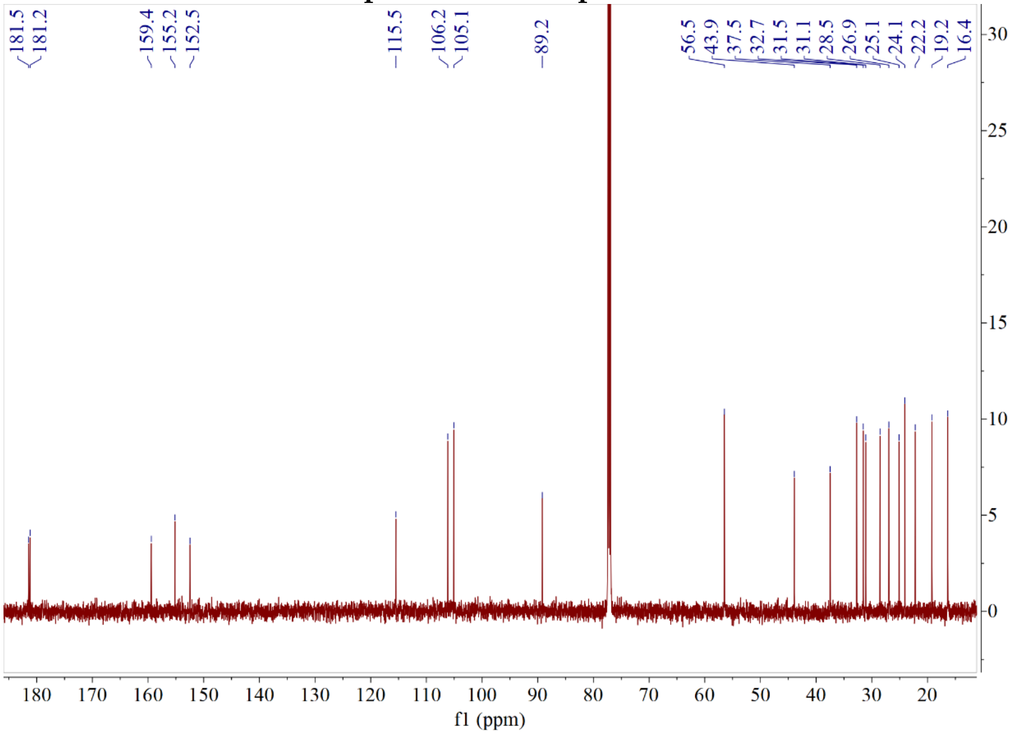

(l) ^{13}C NMR spectrum of compound 18 in $CDCl_3$

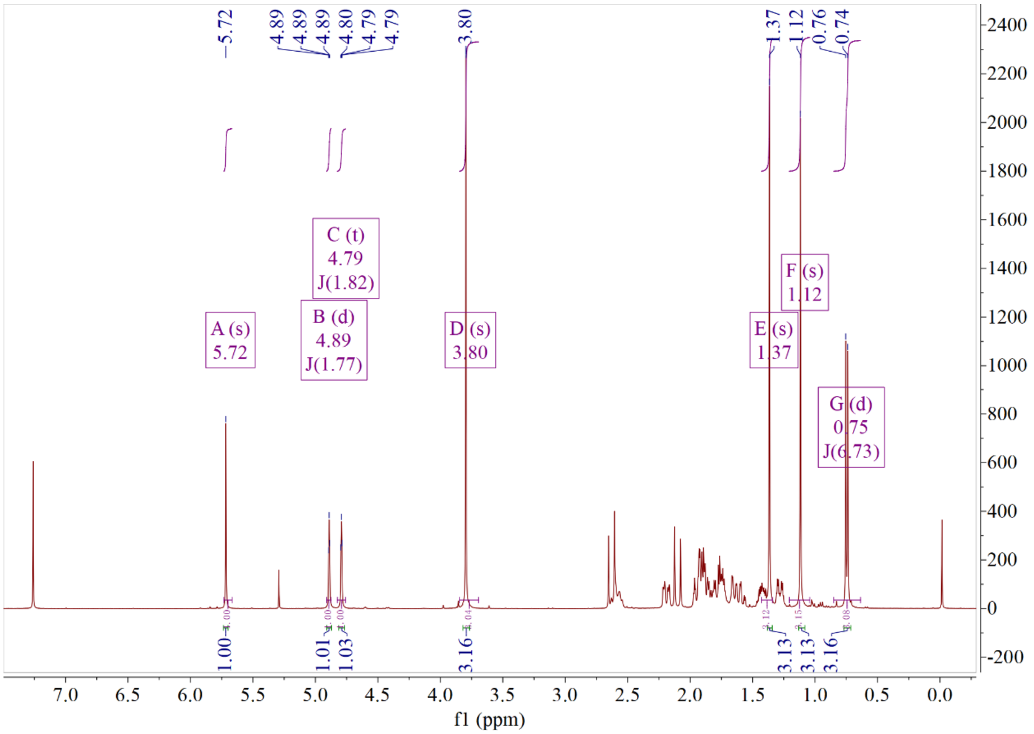

(m) ^{1}H NMR spectrum of compound 19 in $CDCl_3$

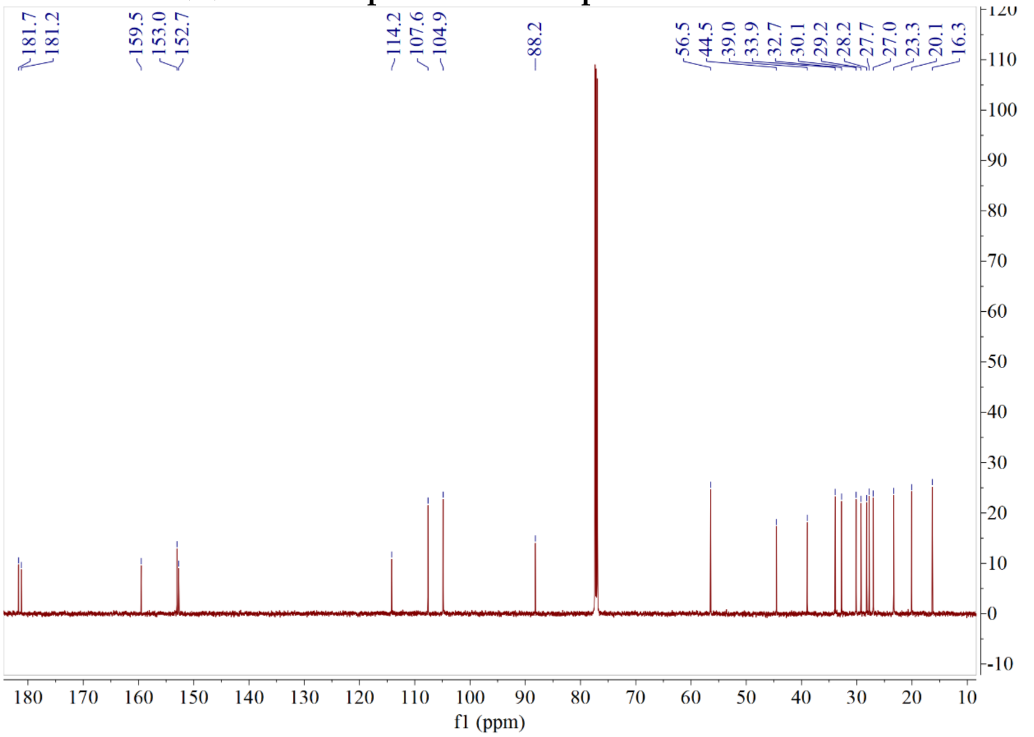

(n) ^{13}C NMR spectrum of compound 19 in $CDCl_3$

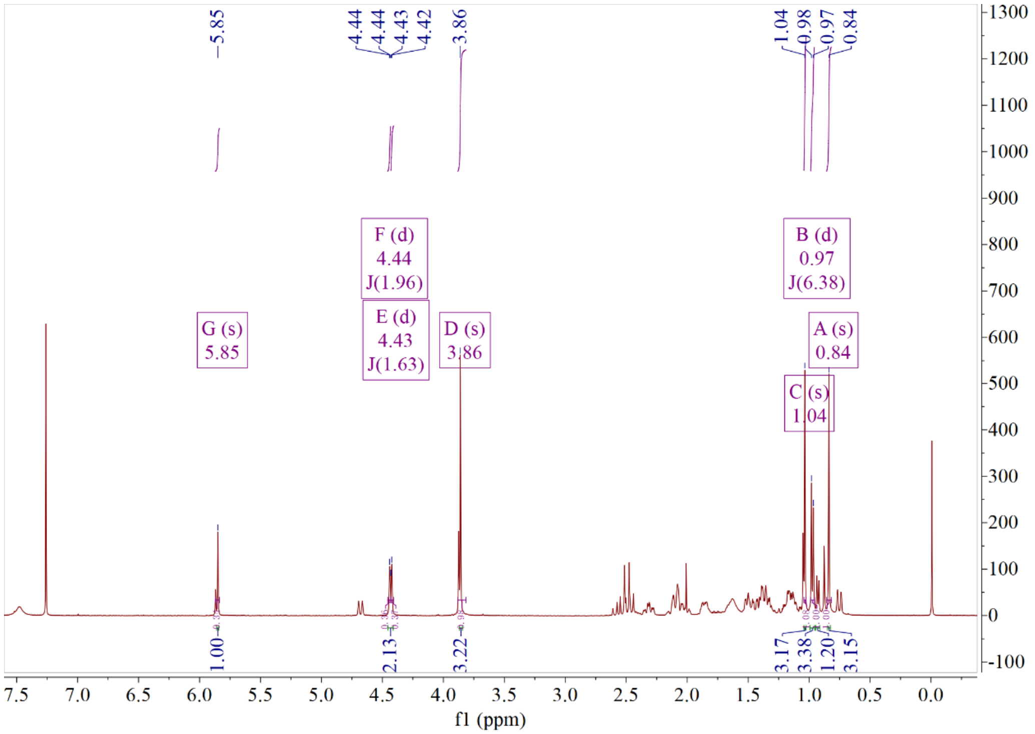

(o) ^{1}H NMR spectrum of compound 20 in $CDCl_3$

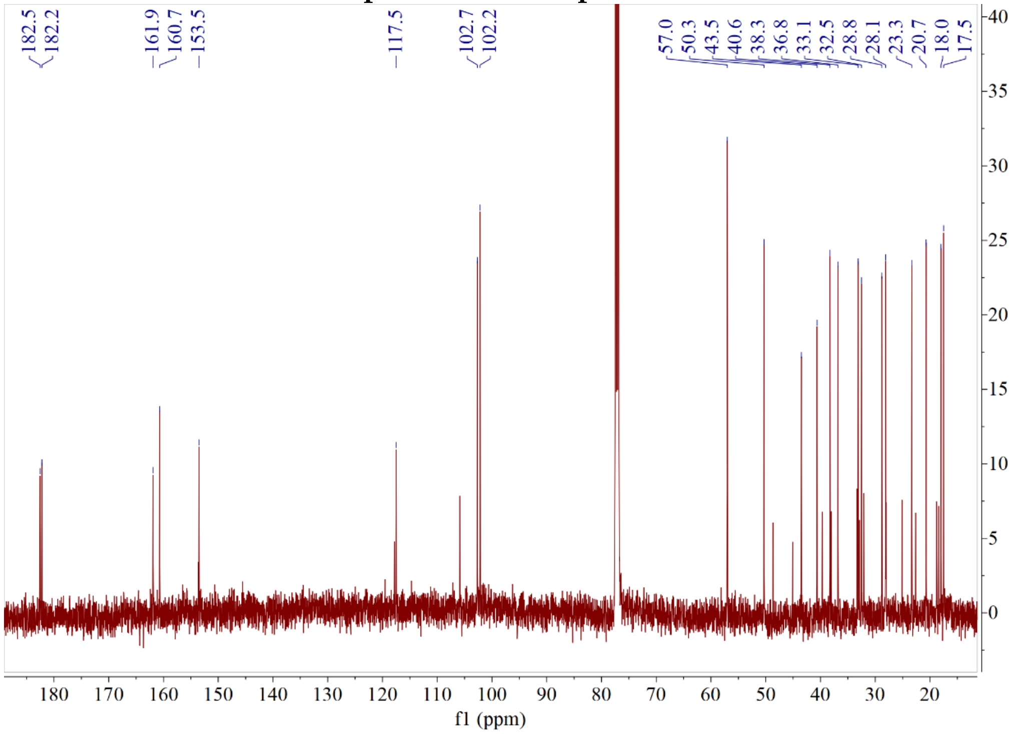

(p) ^{13}C NMR spectrum of compound 20 in $CDCl_3$

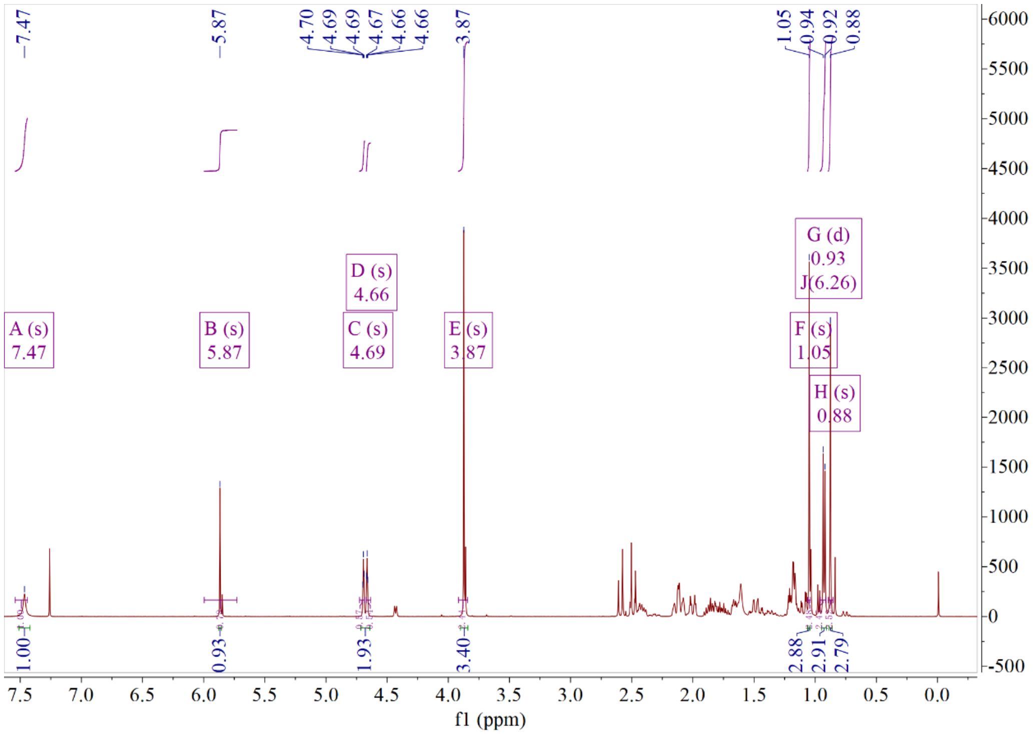

(q) ^{1}H NMR spectrum of compound 21 in $CDCl_3$

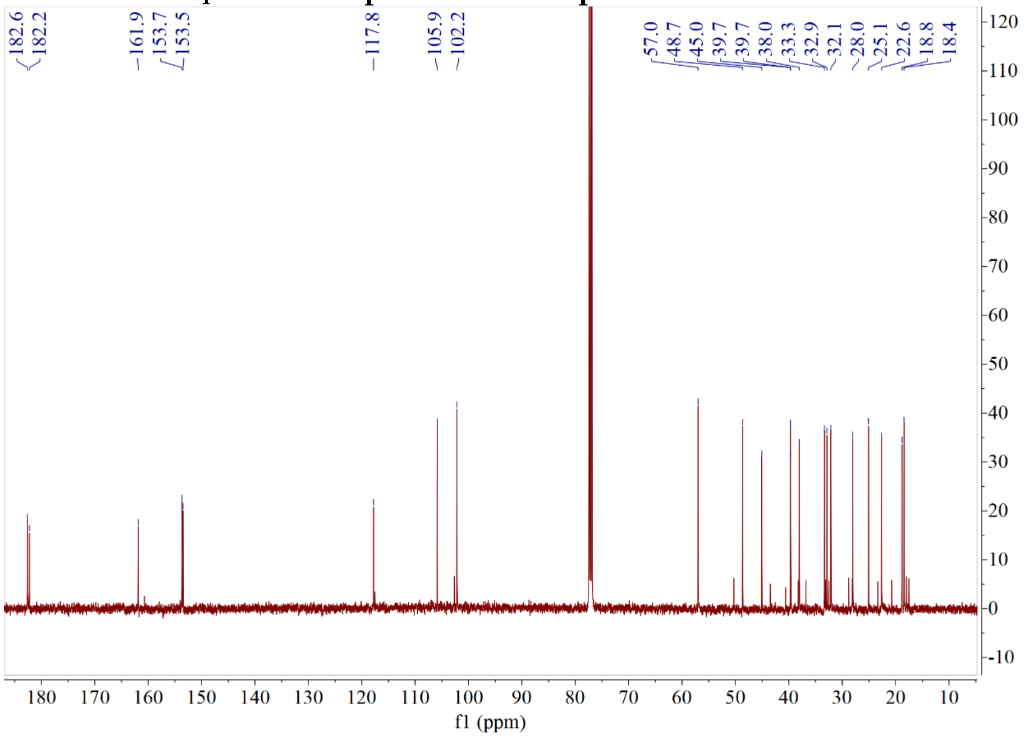

(r) ^{13}C NMR spectrum of compound 21 in $CDCl_3$

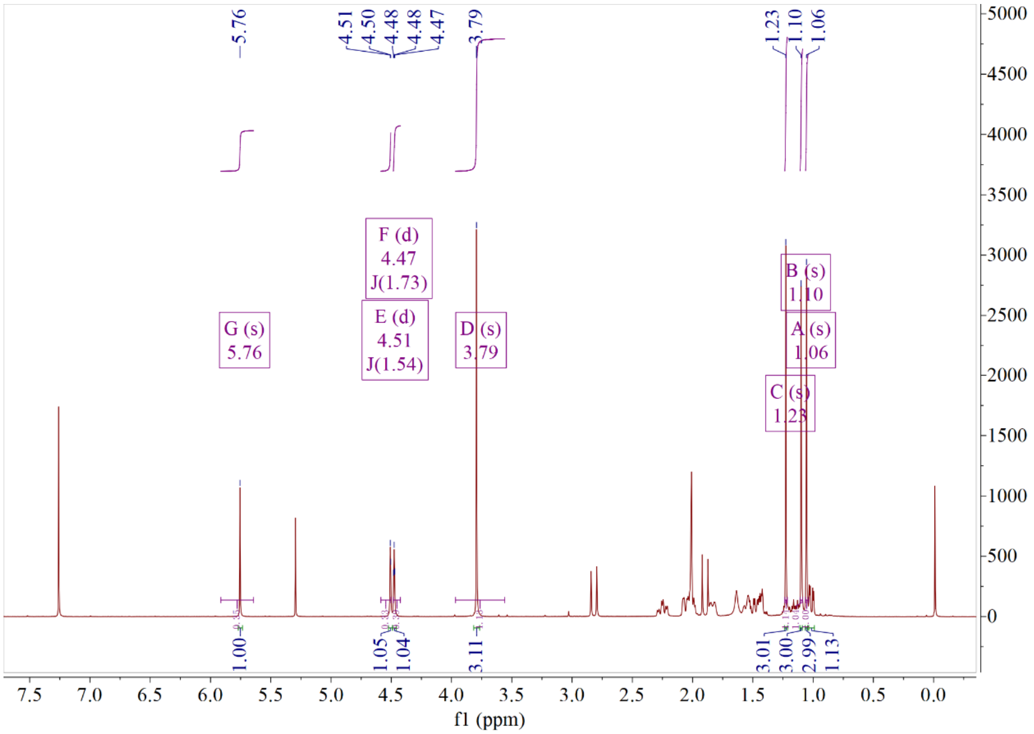

(s) ^{1}H NMR spectrum of compound 22 in $CDCl_3$

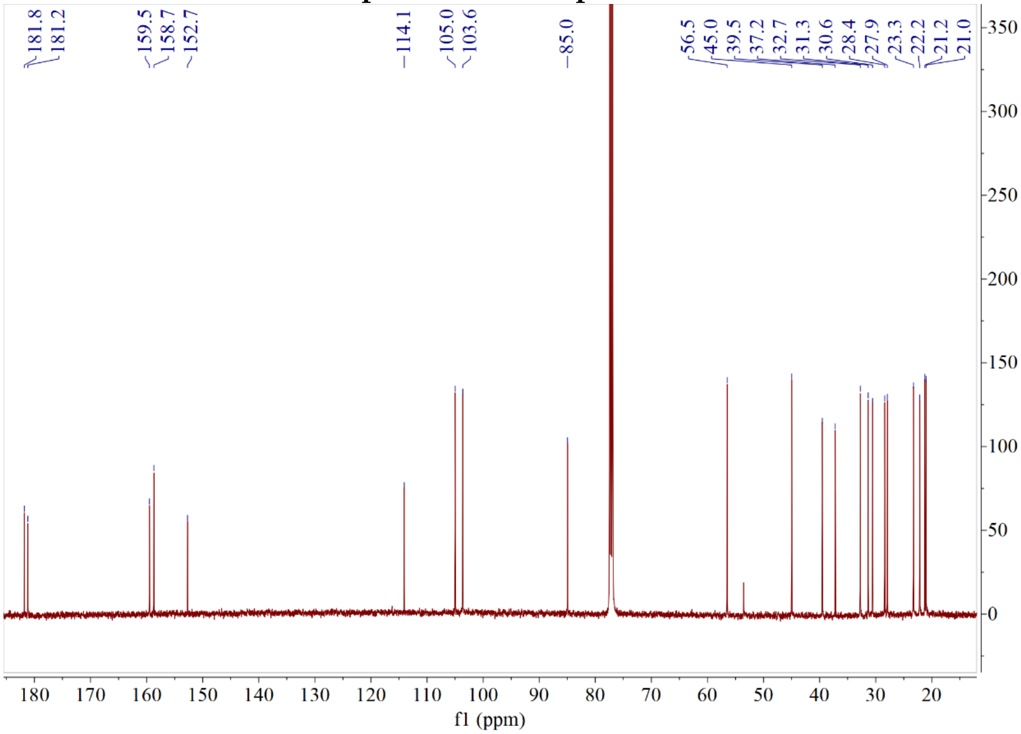

(t) ^{13}C NMR spectrum of compound 22 in $CDCl_3$

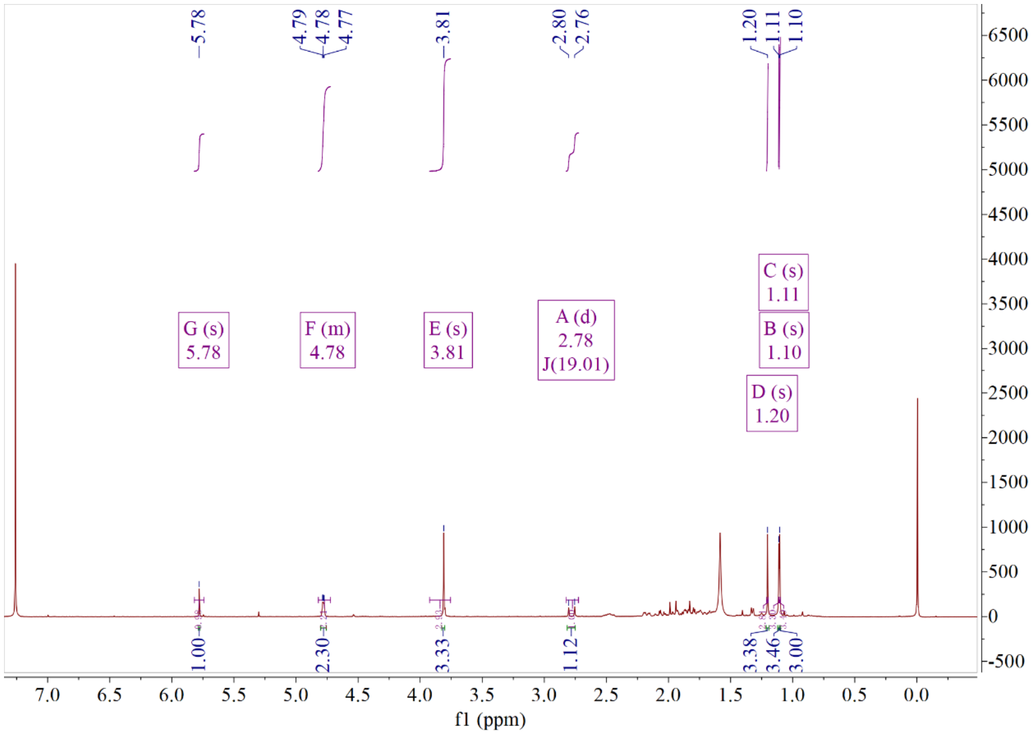

(u) ^{1}H NMR spectrum of compound 23 in $CDCl_3$

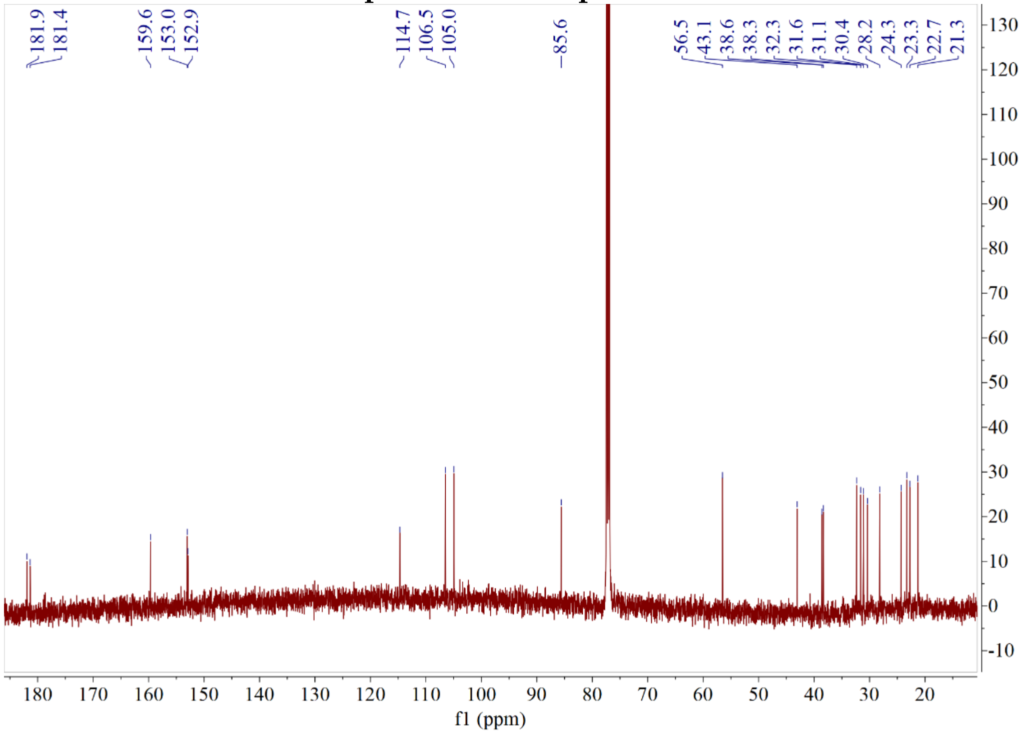

(v) ^{13}C NMR spectrum of compound 23 in $CDCl_3$

Figure S5 ^{1}H and ^{13}C NMR spectra of Compound 12 and 14–23
